# Initial action output and feedback-guided motor behaviors in autism spectrum disorder

**DOI:** 10.1186/s13229-021-00452-8

**Published:** 2021-07-10

**Authors:** Kathryn E. Unruh, Walker S. McKinney, Erin K. Bojanek, Kandace K. Fleming, John A. Sweeney, Matthew W. Mosconi

**Affiliations:** 1 Life Span Institute, University of Kansas, Lawrence, KS USA; 2 Kansas Center for Autism Research and Training (K-CART), University of Kansas, Lawrence, KS USA; 3 Clinical Child Psychology Program, University of Kansas, Lawrence, KS USA; 4 Department of Psychiatry, University of Cincinnati, Cincinnati, OH USA

**Keywords:** Autism spectrum disorder (ASD), Sensorimotor, Eye movement, Precision grip, Lateralization

## Abstract

**Background:**

Sensorimotor issues are common in autism spectrum disorder (ASD), related to core symptoms, and predictive of worse functional outcomes. Deficits in rapid behaviors supported primarily by feedforward mechanisms, and continuous, feedback-guided motor behaviors each have been reported, but the degrees to which they are distinct or co-segregate within individuals and across development are not well understood.

**Methods:**

We characterized behaviors that varied in their involvement of feedforward control relative to feedback control across skeletomotor (precision grip force) and oculomotor (saccades) control systems in 109 individuals with ASD and 101 age-matched typically developing controls (range: 5–29 years) including 58 individuals with ASD and 57 controls who completed both grip and saccade tests. Grip force was examined across multiple force (15, 45, and 85% MVC) and visual gain levels (low, medium, high). Maximum grip force also was examined. During grip force tests, reaction time, initial force output accuracy, variability, and entropy were examined. For the saccade test, latency, accuracy, and trial-wise variability of latency and accuracy were examined.

**Results:**

Relative to controls, individuals with ASD showed similar accuracy of initial grip force but reduced accuracy of saccadic eye movements specific to older ages of our sample. Force variability was greater in ASD relative to controls, but saccade gain variability (across trials) was not different between groups. Force entropy was reduced in ASD, especially at older ages. We also find reduced grip strength in ASD that was more severe in dominant compared to non-dominant hands.

**Limitations:**

Our age-related findings rely on cross-sectional data. Longitudinal studies of sensorimotor behaviors and their associations with ASD symptoms are needed.

**Conclusions:**

We identify reduced accuracy of initial motor output in ASD that was specific to the oculomotor system implicating deficient feedforward control that may be mitigated during slower occurring behaviors executed in the periphery. Individuals with ASD showed increased continuous force variability but similar levels of trial-to-trial saccade accuracy variability suggesting that feedback-guided refinement of motor commands is deficient specifically when adjustments occur rapidly during continuous behavior. We also document reduced lateralization of grip strength in ASD implicating atypical hemispheric specialization.

**Supplementary Information:**

The online version contains supplementary material available at 10.1186/s13229-021-00452-8.

## Introduction

Sensorimotor deficits are common in autism spectrum disorder (ASD), emerge within the first years of life, often prior to social-communication issues [3, 10] and are predictive of worse functional outcomes [9, 68]. While sensorimotor issues repeatedly have been demonstrated in ASD, the majority of existing studies either have approached sensorimotor behaviors as a unitary construct or interrogated component motor processes in isolation. Further, despite evidence that the severity of sensorimotor issues may vary across development [14, 39], few studies have characterized age-dependent differences in select sensorimotor behaviors. As a result, knowledge of the specific motor control processes that are affected in ASD and understanding of their neurodevelopmental substrates remain limited. There is a need to define the nature of sensorimotor issues in ASD, test their variance across development, and determine the relationships among different sensorimotor behaviors in order to better clarify neurodevelopmental mechanisms associated with ASD.

A diverse range of sensorimotor issues has been documented in ASD. In addition to showing reduced coordination of complex lower (e.g., gait; [41]) and upper body movements (e.g., reaching; [16, 17]), and higher rates of dyspraxia [8, 9, 48], individuals with ASD show deficits in sensorimotor behavior that implicate dysfunction of both feedforward motor control mechanisms and sensory feedback processes. Atypical timing and reduced accuracy of initial force output (specific to lower force levels) have been documented during precision gripping in ASD implicating abnormalities in both temporal and spatial (force output) dimensions [6, 7, 74]. Reduced accuracy and velocity of saccadic eye movements also have been documented [38, 61, 65]; however, deficits may not extend across all aspects of oculomotor behavior (e.g., saccade initiation; see [28]) and appear to vary as a function of task difficulty (e.g., [45]). Reduced accuracy of initial force output and visually-guided saccades in ASD implicates deficits in feedforward processes guided by internal models that support output accuracy prior to sensory feedback being available based on afferent delays. Individuals with ASD also show increased variability during sustained force output implicating deficits in multisensory feedback integration and the ability to rapidly translate input error information into precise, reactive motor adjustment [47, 74]. Reduced entropy of individuals’ force time series during sustained precision gripping also has been documented repeatedly in ASD suggesting the ability to integrate the multiple feedback and feedforward processes involved in dynamically adjusting ongoing behavior is compromised. Finally, increased rates of mixed-handedness [11] and reduced differentiation of dominant and non-dominant upper limb movements have been reported in ASD suggesting atypical lateralization of sensorimotor function [53, 54].

Sensorimotor behavioral precision is supported by interacting rapid, feedforward control processes, and slower, feedback control processes involved in processing sensory information. The degree to which these processes interact to support sensorimotor output operates along a continuum that may vary according to different task demands, including the rate at which a behavior must be executed, the difficulty of the task (e.g., force load), and the quality of sensory feedback [63]. For example, visually-guided saccades and initial output of grip force both may rely on consolidated internal action models that are executed via rapid, forward control processes. However, the rate of execution varies between these two processes relative to their effector systems: ballistic oculomotor processes are carried out too rapidly to integrate feedback into ongoing motor plans, while initial grip force is carried out on a relatively protracted timescale because it is executed within the periphery and visual, haptic and proprioceptive feedback processes may contribute to modifications of the output trajectory prior to behavioral endpoints. Therefore, examination of sensorimotor behaviors across effectors, time-scales, and different sensory feedback conditions is critical for characterizing sensorimotor phenotypes in ASD and the extent to which distinct control processes are impacted.

The proposed studies aimed to characterize initial action output and feedback-guided motor behavioral precision in ASD across skelotomotor (hand) and oculomotor systems. We predicted that individuals with ASD would show increased variability of continuous grip force and trial-to-trial saccade accuracy relative to controls, consistent with prior studies from our group and others supporting an over-arching hypothesis that multi-sensory feedback control of motor output is compromised in ASD [35, 47, 74]. We also hypothesized that the accuracy of initial grip force output (primary pulse) and saccades would be reduced compared to controls, in line with prior studies demonstrating impaired feedforward control of rapid motor output in ASD [6, 7, 74]. The complexity of the sustained grip force time series also was examined to test the hypothesis that integration of multiple motor control processes that operate on different time scales is deficient in ASD. Maximum grip strength was measured in dominant and non-dominant hands to test the hypothesis that lateralization of gross motor strength is reduced in ASD relative to controls. Based on prior findings from our group and others [8, 47, 61, 69, 74], we predicted that deficits in sensorimotor function would be associated with core social communication and RRB symptoms of ASD.

## Methods

### Participants

109 individuals with ASD (20 female) and 101 typically developing (TD) controls (28 female) completed clinical testing and three sensorimotor tests, including two precision gripping tests in which force (Force test) or visual gain levels (Gain test) were varied, and a test of visually guided saccades (VGS test). Some individuals did not complete each test based on scheduling issues (Table 1). Individuals were studied at either the University of Illinois at Chicago (UIC) or the University of Texas Southwestern Medical Center (UTSW). Participant groups were similar in terms of sex ratio and handedness. Participants with ASD were recruited through outpatient clinics and community advertisements. Participants completed one of three tests of general cognitive ability selected based on their age. At UIC, participants under 12 years of age completed the Differential Ability Scale, Second Edition (DAS), while participants ≥ 12 years completed the Wechsler Abbreviated Scale of Intelligence (WASI). At UTSW, participants under 6 years of age completed the Wechsler Preschool and Primary Scales of Intelligence, Fourth Edition (WPPSI), while participants ≥ 6 years completed the WASI.

**Table 1:** Participant demographics

	TOTAL		UIC		UTSW	
	Control	ASD	Control	ASD	Control	ASD
Gripping (Force test)						
N	74 (20 F)	76 (11 F)	41 (10 F)	33 (6 F)	33 (10 F)	43 (5 F)
Age	13.8 (6.9)	12.2 (5.3)	14.3 (6.0)	14.6 (5.7)	13.0 (7.9)	10.3 (4.0)
Handedness (R/L)	66/5	61/14	39/2	30/3	30/3	32/11
Verbal IQ	111 (16)	96 (18)*	110 (19)	99 (19)	113 (10)	92 (15)
Nonverbal IQ	107 (15)	100 (18)*	105 (14)	99 (18)	108 (15)	100 (17)
ADOS CSS	–	7.05 (1.87)	–	6.80 (2.00)	–	7.26 (1.75)
Gripping (Gain test)						
N	59 (14 F)	45 (7 F)	40 (10 F)	33 (6 F)	19 (5 F)	12 (2 F)
Age	13.0 (6.6)	13.2 (5.7)	14.7 (6.1)	14.8 (5.7)	9.9 (6.5)	9.1 (3.3)
Handedness (R/L)	50/5	37/6	37/3	30/3	16/3	9/3
Verbal IQ	111 (17)	98 (19)*	110 (19)	100 (20)	113 (12)	86 (14)
Nonverbal IQ	106 (16)	100 (19)	105 (15)	100 (18)	107 (19)	101 (23)
ADOS CSS	–	6.81 (1.85)	–	6.65 (2.02)	–	7.25 (1.21)
Eye movement (VGS test)						
N	82 (19 F)	88 (14 F)	55 (13 F)	61 (12 F)	27 (7 F)	27 (3 F)
Age	14.8 (6.8)	12.8 (5.1)*	14.9 (6.3)	13.6 (5.2)	14.5 (7.8)	11.1 (4.2)
Handedness (R/L)	75/4	74/12	50/3	56/4	25/1	18/8
Verbal IQ	110 (13)	97 (18)*	109 (16)	99 (18)	114 (11)	92 (18)
Nonverbal IQ	106 (13.5)	101 (18)*	104 (12.6)	101 (18)	110 (15)	100 (19)
ADOS CSS	–	6.88 (2.09)	–	6.84 (2.21)	–	7.00 (1.78)
Grip + VGS tests						
N	45 (13 F)	48 (9 F)	25 (6 F)	25 (6 F)	20 (7 F)	23 (3 F)
Age	15.0 (6.9)	13.3 (5.3)	15.5 (6.1)	15.0 (5.6)	14.5 (7.8)	11.3 (4.1)

*ASD* autism spectrum disorder, *F* female, *ADOS* Autism Diagnostic Observation Schedule, *CSS* Calibrated Severity Score, *MVC* maximum voluntary contraction, *VGS* visually-guided saccade; Mean(SD). *group differences at *p* < .05

ASD diagnoses were confirmed using the Autism Diagnostic Inventory-Revised (ADI-R; [37]) and the Autism Diagnostic Observation Schedule—Second Edition (ADOS, [36]), which were used to evaluate participants based on Diagnostic and Statistical Manual of Mental Disorders (DSM) criteria. These procedures were performed by study team members who had reached research and within-site reliability on these measures. Participants assessed prior to 2013 were diagnosed according to DSM-IV TR, and all participants studied after publication of DSM-5 were diagnosed according to updated criteria. Participants with ASD were excluded for known genetic or metabolic disorders associated with ASD (e.g., fragile X syndrome, Tuberous sclerosis). Handedness was determined using self-report.

General exclusion criteria included self- or caregiver report of any history of substance dependence or abuse within the previous six months, history of non-febrile seizures or head trauma with loss of consciousness, complications during pregnancy, delivery, or perinatal period, or current use of medications known to interfere with sensorimotor behavior including stimulants, antipsychotics, anticonvulsants or benzodiazepines [59]. TD controls were excluded if they had a known lifetime history of psychiatric or significant medical disorder, had a family history of a major psychiatric disorder in their first-degree relatives, or a history of ASD in first or second-degree relatives. Participants refrained from caffeine, nicotine, and alcohol on the day of testing and over-the-counter drugs with sedating properties (e.g., cold medicine) within 12 h of testing. Written informed consent was obtained from all participants, with assent and parental consent obtained for minors. Study procedures were approved by the local Institutional Review Boards.

### Precision grip testing

**Fig. 1:**
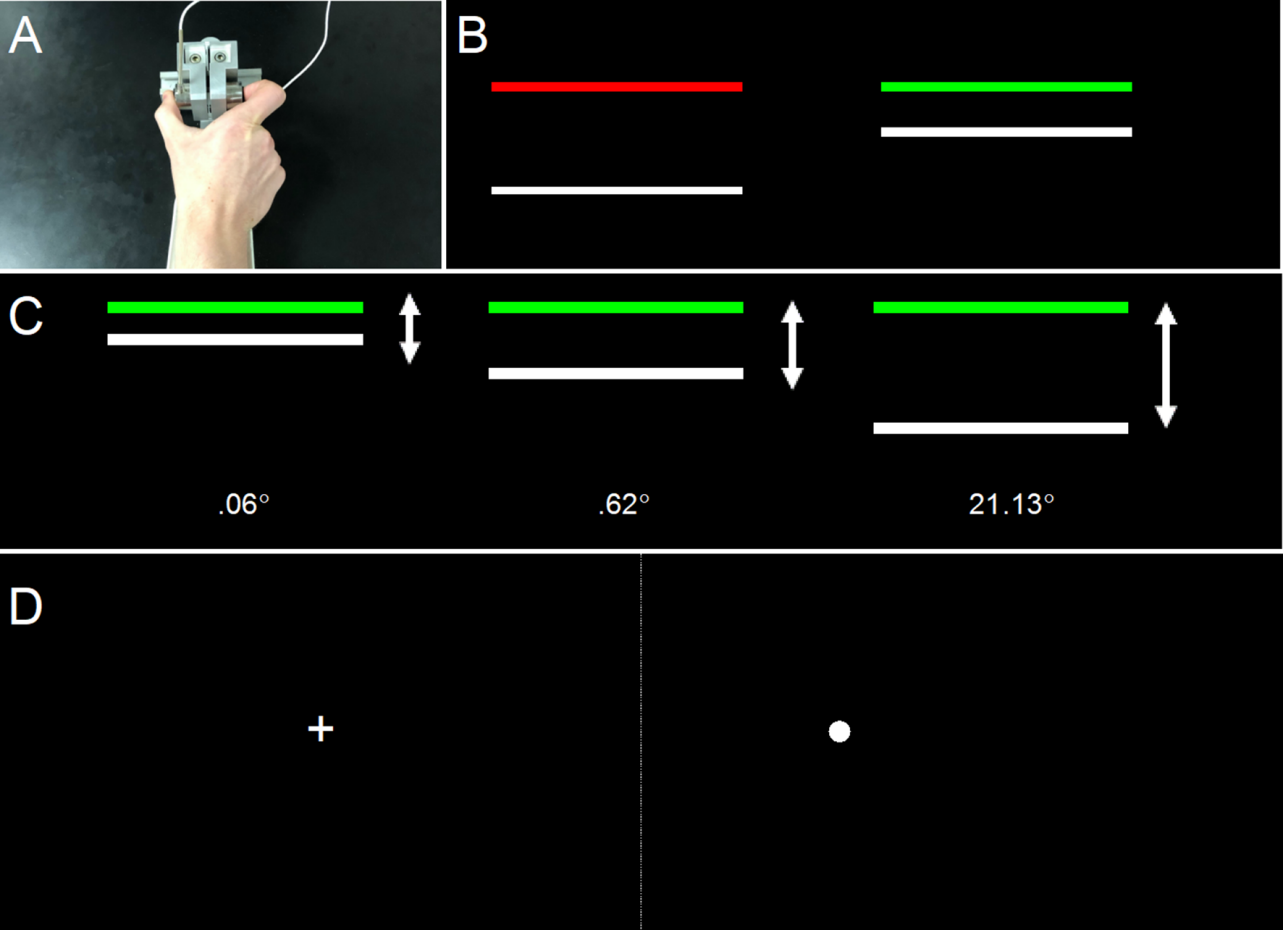
**A** Precision grip load cell apparatus. **B** Precision grip stimuli. Participants viewed a red TARGET bar and white FORCE bar. Once the TARGET bar turned green, participants pressed the load cells to bring the white FORCE bar up to the level of the green TARGET bar. **C** Precision grip visual gain manipulation. The amount of visual feedback was either degraded (left: 0.06°) or amplified (rightmost: 21.13°). **D** Visually guided saccade stimuli. Participants viewed crosshairs and made a saccade to the left (− 12°; depicted) or right (+ 12°)

Stimuli were presented on a 102 cm monitor with 1366 × 768 resolution and 120 Hz refresh rate. Participants were seated in a darkened room 53 cm from the display screen with their elbow at 90° and arm in a relaxed position in a custom arm brace designed to keep the individual’s arm steady throughout testing. Participants used their thumb and index finger to press against two opposing precision load cells (ELFF-B4-100N; Entran) 1.27 cm in diameter secured to a custom grip device attached to the arm brace (Fig. 1A). A Coulbourn (V72-25) resistive bridge strain amplifier received analog signal from the load cells. Data were sampled at 120 Hz with a 16-bit analog-to-digital converter (DI-720; DATAQ Instruments) and converted to Newtons using a calibration factor derived from known weights before the study [47].

Before testing, each participant’s maximum voluntary contraction (MVC) was calculated separately for each hand using the average of the maximum force output during three trials of three seconds each. Trials alternated between left and right hands with at least 15 s of rest between each hand. The participant was instructed to “press [on the transducer] as hard as you can when the computer reads GO using only your thumb and pointer finger.” Participants were continuously monitored by the research administrator to ensure only the thumb and forefinger were in contact with the force transducer. If additional fingers were used during a trial, it was flagged for exclusion and an additional trial was administered.

During testing, participants viewed a horizontal white bar (FORCE) on a black screen that moved upward when force was applied to the load cells (Fig. 1B). A static TARGET bar turned from red to green to indicate the beginning of each trial and participants were instructed to (1) press the load cells as quickly as possible when the TARGET bar turned green and (2) hold the FORCE bar steady at the level of the TARGET bar. At UIC, trials were 15 s and alternated with 15 s of rest. At UTSW, trials were 8 s and alternated with 8 s of rest.

Participants were administered two precision gripping tests (Fig. 1). First, in order to determine the extent to which force load affected precision force control, participants were administered a *Force Test* in which they completed three trials with each hand at 15, 45, and 85% of their MVC. Visual angle was held constant at 0.62°. In order to determine the impact of changing the quality of visual feedback on precision force control, participants were administered a *Gain Test* in which the vertical distance the FORCE bar moved in response to changes in force output was varied (Fig. 1C). For example, in the smallest visual gain condition, the force bar moved 0.06 mm per 1 N increase in force. Participants completed three trials with each hand at 0.06, 0.62, and 21.13° of visual angle, consistent with our prior studies [47, 74]. Force output was held constant at 15% of MVC. Precision grip test experiment order was counterbalanced across participants.

As described previously, multiple training procedures were used to ensure participants understood task demands for the precision gripping tests [47, 61, 74]. First, the examiner presented visual slides that showed participants what the task would look like and what they should be doing during the test. The following instructions were given both visually and orally to the participant: “First, you will see one red bar and one white bar on the screen. Whenever the top bar is red, make sure you don’t press the buttons [force transducers]. Next, the red bar will turn green. Press on the buttons so that the white bar reaches the green bar as fast as possible. Keep it there until the green bar turns red. Remember, the harder you press, the higher the white bar will go, so try to keep the white bar as close to the green bar as possible and press as quickly as possible.” Second, the participant completed at least two practice trials prior to beginning the task. During these practice trials, the administrator checked for task compliance including: (1) use of only the thumb and forefinger to press the transducer, (2) exerting appropriate force for the white bar to reach and stay close to the level of the green target bar, and (3) pressing for the entire duration of the trial. If the participant did not show evidence of their ability to comply with each of these goals, the instructions were re-introduced and additional practice trials were administered.

### Oculomotor testing

Participants were tested in a dark room, seated 60 cm from a 102 cm anti-glare LCD monitor (resolution: 1920 × 1060) with head stabilized using a chin-rest to minimize movement (UIC refresh rate = 120 Hz; UTSW refresh rate = 60 Hz). At UIC, eye movements were recorded using infrared (IR) sclera-reflection sensors mounted on spectacle frames (Model 310, Applied Science Laboratories, Bedford, MA) using a 12-bit A/D converter (500 Hz; DI-720 from Dataq Instruments, Akron, OH). Blinks were monitored using direct current electro-oculography (EOG; Grass Neurodata 12 Acquisition System; Astro-Med, Inc., West Warwick, RI). EOG electrodes were placed above and below the left eye and were linked to an AC-coupled bioamplifier. At UTSW, eye movements were recorded using an infrared, binocular camera-based eye tracking system (500 Hz; EyeLink II, SR Research Ltd., Canada). Across both sites, participants performed a nine-point calibration before each block of trials.

During the visually guided saccade test (Fig. 1D), visual stimuli subtending 0.5° of visual angle were presented in the horizonal plane at eye level. Following the presentation of a central fixation appearing for 1.5–2.5 s (varied randomly), a peripheral target was presented for 1.5 s at ± 12°. Fifteen trials were administered for each location (30 total trials); location order varied pseudo randomly. Participants were instructed to look to the target as quickly as possible.

### Data processing

#### Precision grip data

Force data were analyzed with a custom algorithm and scoring program developed previously by our group using MATLAB (MathWorks; [74]). For data from UIC (15 s trials), the first two seconds and the last second of each force trace were excluded from analyses due to variability in the rate at which individuals reached the target force and terminated the trial [60] and trials for which participants produced fewer than 6 s of continuous force data were excluded from analyses. For data from UTSW (8 s trials), the first second and last second of each force trace were excluded from analyses and trials for which the participants produced fewer than 5 s of continuous force data were excluded from analyses. Across both sites, trials also were excluded if the mean force exceeded twice the target force or was less than half of the target force. Force data were linearly detrended to account for systematic changes in the mean force over the duration of the trial. Data from each trial were visually inspected offline to ensure proper calibration of load cells and task compliance (e.g., pressing during rest periods or failure to press for the duration of the trial) and scored without examiner knowledge of participant characteristics (e.g., age or diagnostic status).

To assess rapid force control, the initial (i.e. primary) pulse of the force trace was examined during 15% MVC trials as previously reported [47, 74]. Trials using higher force levels were not examined based on the rationale that rapid force processes occur over a brief very duration. The primary pulse at 15% MVC is smaller in amplitude relative to higher force levels and therefore minimizes the amount of time over which the primary pulse can occur. Reaction time and accuracy of the primary pulse were tested. Reaction time reflected the difference between trial onset and onset of the primary pulse. Onset of the primary pulse was defined as the point at which the rate of force increase exceeded 5% of the peak rate of onset and remained at this level for at least 100 ms. Accuracy was calculated as the force at the offset of the primary pulse divided by the target force using methods described previously [74, 75]. Primary pulse offset was defined at the first zero-crossing of the velocity trace, acceleration trace, or jerk trace (third derivative) following the peak velocity, whichever comes first [74]. Variability and entropy of the sustained phase of the force trace also was examined during all conditions of the force (15, 45, 85% MVC) and gain (0.06, 0.62, 21.13 degree) tests. Force variability was defined as the standard deviation (SD) of the linearly detrended sustained force time series. To account for differences in variability due to individual differences in force output, the coefficient of variation (CoV) was calculated by dividing the force SD by the mean force level for each trial. Approximate entropy (ApEn) was calculated to examine the time-dependent structure of the force series [56, 64, 71]. ApEn values range from 0–2 and indicate the predictability of future values in a time series given a set of previous values, with lower numbers corresponding to more predictable data and higher numbers corresponding to more irregular, or complex, data.

#### Oculomotor data

Digital finite impulse response filters with non-linear transition bands were applied with a gradual transition band (from pass to no pass) between 20 and 65 Hz for velocity and position data, and between 30 and 65 Hz for acceleration data. Data from each trial were visually inspected offline and scored without examiner knowledge of participant characteristics (e.g., age or diagnostic status). Trials were calibrated independently using fixation data from central and peripheral target locations. Each trial was manually calibrated by marking the stable center fixation prior to trial onset, and at the target location after the participant acquired the peripheral target. Trials were evaluated for signal drift and head movement and re-calibrated using within-trial data from fixation of targets of interest as we have done previously [61]. Saccade onset and offset were marked where velocity exceeded or fell back below 30 deg per second, respectively. Trials with latencies < 70 ms were considered anticipatory and were not included in analyses. Trials were excluded if a blink occurred 100 ms prior to stimulus presentation or prior to the end of the primary saccade.

Saccade latency and gain and their trial-to-trial variability were examined. Saccade latency was defined as the difference between peripheral target onset and saccade initiation. Saccade gain was defined as the ratio of the saccade amplitude to the target amplitude [44, 46, 61], with values below 1 indicating saccade hypometria (saccade amplitude does not reach the target location) and values greater than 1 indicating saccade hypermetria (saccade amplitude exceeds the target location). Variability of saccade latency and variable of saccade gain were defined as their SD across trials.

### Clinical measures

In order to assess the severity of individuals’ ASD symptoms, we examined the calibrated severity score (CSS) of the ADOS. The ADOS is a semi-structured assessment of social-communicative abnormalities and restricted, repetitive behaviors characteristic of ASD. The CSS is computed based on raw total percentiles that allow for comparison of symptom severity across ADOS modules selected based on age and language level [18]. Diagnostic algorithm scores from the ADI-R also were used to assess severity of social interaction and communication abnormalities and repetitive or stereotyped patterns of behavior. To examine subtypes of repetitive behavior, including repetitive sensorimotor behaviors, insistence on sameness, rituals, compulsions, and restricted interests, the Repetitive Behavior Scale-Revised (RBS-R; [31]) also was used. Across all clinical measures, higher scores reflect greater symptom severity.

### Statistical analyses

To determine whether sensorimotor behavior differed according to diagnostic group, age, or laterality (dominant vs. non-dominant hand for precision grip; rightward vs leftward for saccades), separate linear mixed effect analyses were conducted for each dependent variable of interest [2, 29]. Level one (within-subjects) predictors for the precision grip test included *condition* (Force test: 15, 45, or 85% MVC; Gain test: 0.06, 0.62, 21.13 degrees of visual angle) and *hand tested* (dominant vs. nondominant). Level one predictors for the eye movement test included *target location* (+ vs. − 12 deg). Level two (between-subjects) predictors were the same for grip and eye movement tests and included *age* and *diagnostic group*. Location of data collection (UIC or UTSW) was included as a level two covariate of no interest. For primary analyses, sex also was included as a level two covariate of no interest given that our sample of females was not sufficient for estimating sex or sex × group effects. Results from exploratory models including sex as a level two (between-subjects) predictor are reported in the Additional file 2.

To limit the number of statistical analyses performed and maintain parsimonious models, as is consistent with best-practice recommendations [42], initial models included only three-way interactions testing a priori hypotheses and their nested two-way interactions. To identify the best-fitting models, predictors were iteratively removed and model fit was compared between the previous and subsequent models using log likelihood ratio tests [22]. Predictors that significantly improved model fit (*p* < 0.05) were retained in the final model. Age was centered around the grand mean and categorical predictors were reference coded. Based on this scheme, model intercepts can be interpreted as follows. Force test: 15% MVC performance for an average aged (13.1) male, healthy control, using their dominant hand; Gain test: 0.06° visual angle performance for an average aged (13.1) male, healthy control, using their dominant hand; VGS: right target (+ 12°) performance for an average aged (13.7) male, healthy control. Main effects and interaction results are reported relative to these baseline reference values. Significant 3-way interactions involving age were followed up with regression analyses testing relevant 2-way interactions and simple effects. In the absence of a significant 2-way interaction, simple effects were interpreted and reported.

Mixed effects modeling was conducted using the *lme4* package [2], reported model statistics were calculated using the *lmerTest* package [30], and linear regression models were conducted using the base R stats package within R version 3.6.3. Simulations have demonstrated that maximum likelihood estimators used to evaluate fixed effects in linear models are generally robust to violations of assumptions, including non-gaussian error distributions [27, 76], particularly when sample size exceeds 50 [40]. Based on these simulations and the structure of our data, our reported model statistics use traditional log-likelihood estimates of standard errors which allow for calculation of readily-interpretable ANOVA statistics. All models are reported in the Additional file 2.

Pearson correlations were computed to examine the relationships between dependent variables within and across tasks and Fisher’s r-to-z transformations [4] were used to compare the strength of these relationships between groups. Spearman correlations (SPSS version 27) were computed to examine the relationships between sensorimotor variables that were different between groups and ADOS calibrated severity scores, ADI diagnostic algorithm scores, and RBS-R repetitive behavior subscale (stereotyped motor movements, self-injurious behavior, rituals, compulsions, insistence on sameness, and restricted interests) and total scores. Clinical correlation analyses included individuals with ASD only. For all correlation analyses, the Benjamini–Hochberg method was used to control for Type I error.

## Results

### Maximum voluntary contraction

**Fig. 2:**
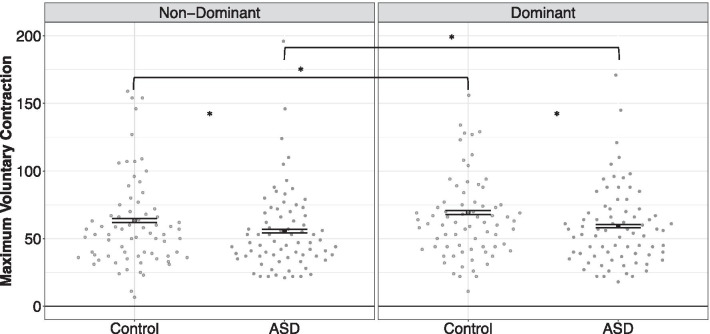
MVC was reduced in ASD participants relative to TD controls across both dominant and non-dominant hands. MVC differences between dominant and non-dominant hands were reduced in individuals with ASD relative to controls. Error bars reflect standard error of the mean. *p < *.05*

Individuals with ASD showed reduced MVCs compared to TD controls, and the severity of these differences varied as a function of hand tested (Fig. 2; group x hand tested: *F*_1,2103_ = 54.00, *p* < .001). MVC was reduced in ASD relative to controls to a greater degree in the dominant hand (ASD vs. control: *t*(1089) = 5.33, *p* < .001, *d* = .32) than in the non-dominant hand (ASD vs. control: *t*(1082) = 3.99, *p* < .001; *d* = .24). MVCs were greater for dominant compared to non-dominant hands, though differences in MVC between dominant and non-dominant hands were smaller for individuals with ASD (dominant vs. non-dominant: *t*(1147) = −2.09, *p* = .03; *d* = .13) relative to controls (dominant vs. non-dominant: *t*(1098) = −2.90, *p* = .003; *d* = .18). MVC increased as a function of age across participants (age: *F*_1,144_ = 175.74, *p* < .001; = 38.12, *t* = 34.56, *p* < .003, *R2* = .35). See Fig. 2.

### Reaction time of initial action output

#### Primary pulse reaction time (15% MVC)

Reaction time of the primary pulse did not vary as a function of group (*F*_1,114_ = 1.07, *p* = 0.30), age (*F*_1,111_ = 1.85, *p* = 0.18), or hand-tested (*F*_1,468_ = 1.12, *p* = 0.29).

#### Saccade latency

Latency did not vary as a function of group or direction, but was inversely associated with age (age: *F*_1,169_ = 20.77, *p* < 0.001; *β* = − 2.02, *t* = − 6.23, *p* < 0.001, *R*^*2*^ = 0.09). These results indicate that increased age was associated with decreased latency across participants.

#### Trial-To-Trial variability of saccade latency

A significant 3-way interaction indicated the association between age and trial-to-trial variability of saccade latency varied as a function of group and direction (Additional file 4: Fig. S1; age × group × direction: *F*_1,168_ = 4.58, *p* = 0.03). Latency variability decreased with age in both groups for leftward saccades (− 12 degree trials, group × age: *β* = − 0.32, *t* = − 0.80, *p* = 0.42). For rightward saccades, control participants again showed age-related reductions in latency variability (*β* = − 0.72, *t* = − 3.16, *p* = 0.002), while individuals with ASD did not (*β* = − 0.03, *t* = − 0.09, *p* = 0.93).

### Accuracy of initial action output

#### Primary pulse accuracy (15% MVC)

Primary pulse accuracy did not vary as a function of group (*F*_1,100_ = 1.84, *p* = 0.18), age (*F*_1,97_ = 3.52, *p* = 0.06), or hand-tested (*F*_1,428_ = 0.10, *p* = 0.75).

#### Saccade gain

**Fig. 3:**
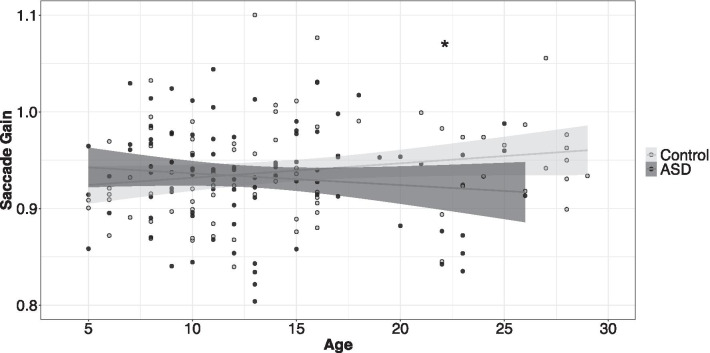
Increased age was associated with increased saccade accuracy in controls and reduced saccade accuracy in individuals with ASD. Error bars reflect standard error of the mean. *slopes differ between groups

Individuals with ASD showed reduced saccade gain relative to controls, though this difference varied as a function of age (Fig. 3; group × age: *F*_1,164_ = 6.69, *p* = 0.01). Controls showed greater saccade accuracy (gain values closer to 1) with increased age (*β* = 0.001, *t* = 3.69, *p* < 0.001, *R*^*2*^ = 0.01), while individuals with ASD showed relative reductions in saccade accuracy with increased age (*β* = − 0.002, *t* = − 3.55, *p* < 0.001, *R*^*2*^ = 0.01).

### Variability of feedback-guided motor adjustments

#### Force test: grip force coefficient of variation (CoV)

**Fig. 4:**
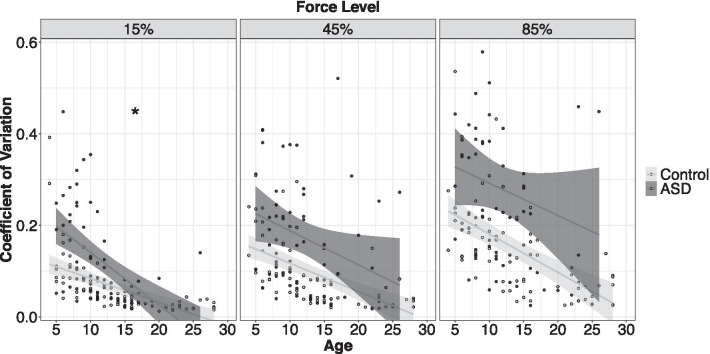
Age-associated decreases in force variability were stronger in ASD relative to controls at low force. At medium and high force, force variability was increased in ASD relative to controls, but age-associated improvements in variability were similar across groups. Error bars reflect standard error of the mean. *slopes differ between groups

Individuals with ASD showed increased force CoV compared to controls, though group differences varied as a function of age and target force level (Fig. 4; age × group × MVC: *F*_2,1966_ = 12.39, *p* < 0.001). This three-way interaction reflected the findings that CoV decreased as a function of age more strongly in ASD relative to controls at 15% MVC (age × group: *β* = − 0.005, *t* = − 4.91, *p* < 0.001, *R*^*2*^ = 0.22) but not at 45% (age × group: *β* = − 0.001, *t* = − 0.66, *p* = 0.50) or 85% MVC (age × group: *β* = − 0.001, *t* = − 0.13, *p* = 0.89). CoV was elevated in ASD relative to controls at 45% (group: *β* = 0.07, *t* = 2.96, *p* = 0.003, *R*^*2*^ = 0.13) and 85% MVC (group: *β* = 0.09, *t* = 2.82, *p* = 0.005, *R*^*2*^ = 0.16).

#### Gain test: grip force coefficient of variation (CoV)

**Fig. 5:**
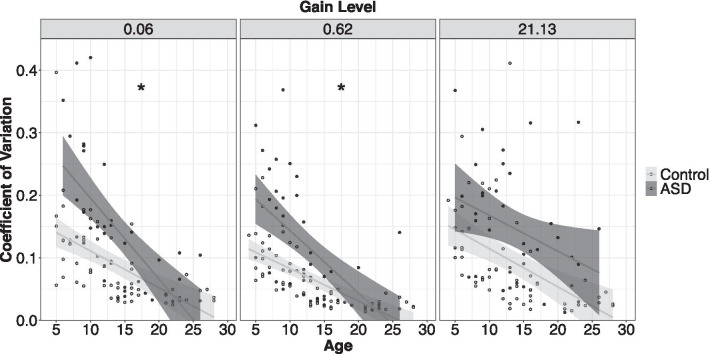
Age-associated reductions in force variability were stronger in ASD relative to controls at low and medium gain. At high gain, force variability was increased in ASD relative to controls, but age-associated reductions in variability were similar across groups. Error bars reflect standard error of the mean. *slopes differ between groups

Individuals with ASD showed increased force CoV compared to controls, though the severity of this difference varied as a function of age and gain level (Fig. 5; age × group × gain: *F*_2,1450_ = 5.62, *p* = 0.004). Age-associated reductions in CoV were stronger in ASD relative to controls at low (age × group: *β* = − 0.01, *t* = − 6.07, *p* < 0.001, *R*^*2*^ = 0.29) and medium gain (age × group: *β* = − 0.005, *t* = − 3.41, *p* < 0.001, *R*^*2*^ = 0.23), but not at high gain (age × group: *β* = − 0.002, *t* = − 0.97, *p* = 0.33). Force CoV was elevated in ASD relative to controls at high gain (group: *β* = 0.13, *t* = 3.54, *p* < 0.001, *R*^*2*^ = 0.17).

#### Saccade gain variability

Trial-to-trial variability of gain did not vary as a function of group (*F*_1,163_ = 1.14, *p* = 0.28) or direction (*F*_1,163_ = 0.94, *p* = 0.33), but decreased as a function of age (age: *F*_1,166_ = 14.78, *p* < 0.001; *β* = − 0.002, *t* = − 3.33, *p* < 0.001, *R*^*2*^ = 0.03).

### Time-dependent structure of force output

#### Force test: grip force approximate entropy (ApEn)

**Fig. 6:**
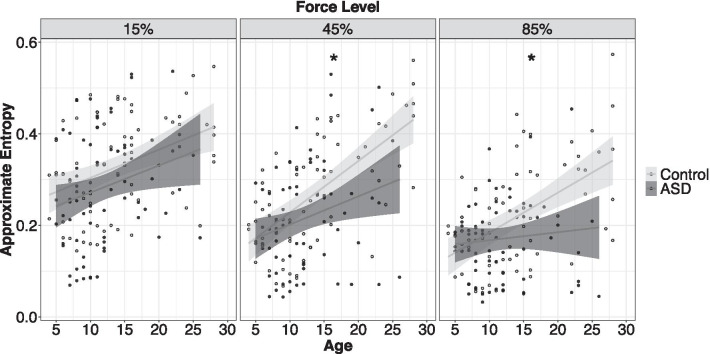
Age-associated reductions in force variability were stronger in ASD relative to controls at low and medium gain. At high gain, force variability was increased in ASD relative to controls, but age-associated reductions in variability were similar across groups. Error bars reflect standard error of the mean. *slopes differ between groups

Individuals with ASD showed reduced force ApEn compared to controls, though the severity of this difference varied as a function of age and target force level (Fig. 6; age × group × MVC: *F*_2,1994_ = 6.45, *p* = 0.002). Increased age was more strongly associated with increased ApEn in controls relative to individuals with ASD at 45% (age × group: *β* = − 0.005, *t* = − 3.10, *p* = 0.002, *R*^*2*^ = 0.25) and 85% MVC (age × group: *β* = − 0.004, *t* = − 2.35, *p* = 0.02, *R*^*2*^ = 0.18), but not at 15% MVC (age × group: *β* = − 0.001, *t* = − 0.72, *p* = 0.47).

#### Gain test: grip force approximate entropy (ApEn)

**Fig. 7:**
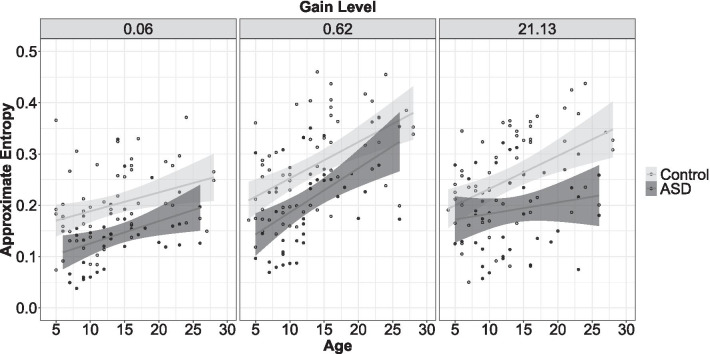
Age-associated improvements in ApEn were similar across groups at low and medium gain. At high gain, individuals with ASD did not demonstrate age-associated improvements in ApEn. Error bars reflect standard error of the mean

Individuals with ASD showed reduced force ApEn compared to controls, though the severity of this difference varied as a function of age and gain level (Fig. 7; age × group × gain: *F*_2,1401_ = 3.49, *p* = 0.03). Post-hoc regression analyses suggested that age-associated gains in ApEn showed trend-level reductions at high gain for ASD compared to controls (age × group: *β* = − 0.003, *t* = − 1.90, *p* = 0.06) but were similar across groups at low (age × group: *β* = − 0.001, *t* = − 0.40, *p* = 0.68) and medium gain (age × group: *β* = − 0.001, *t* = 0.97, *p* = 0.33). Group differences in ApEn also varied according to age and hand tested (age × group × hand tested: *F*_1,1404_ = 6.06, *p* = 0.01; Additional file 4: Fig. S2). This three-way interaction indicated that age-associated increases in ApEn were stronger in controls relative to ASD for the non-dominant (age × group: *β* = − 0.001, *t* = − 2.01, *p* = 0.04, *R*^*2*^ = 0.14) but not the dominant hand (age × group: *β* = 0.000, *t* = 0.97, *p* = 0.33).

### Inter-correlation of sensorimotor behaviors

To determine the degree to which sensorimotor behaviors are distinct or co-segregate within individuals, we examined associations between initial action output and feedback-guided motor adjustments and their associations with reaction time and force entropy. To aid interpretability of these associations, primary pulse accuracy and saccade gain were centered around zero and transformed to the absolute value to indicate the absolute error of the variable.

#### Reaction time—initial action output

Force reaction time was not associated with saccade latency or variability of saccade latency (Table 2). Saccade latency and latency variability also were not associated. Force reaction time, saccade latency, and trial-wise variability of saccade latency were not associated with primary pulse error. Force reaction time was not associated with saccade error. For individuals with ASD, increased saccade latency was associated with increased saccade error. For control participants, trial-to-trial variability of saccade latency was associated with increased saccade error.

**Table 2:** Correlations between reaction time and initial action output

	Primary pulse reaction time	Saccade latency	Latency variability
Primary pulse absolute error			
Control			
Pearson correlation	− 0.042	− 0.068	− 0.068
*p*-value (2-tailed)	0.757	0.659	0.656
*N*	56	45	45
ASD			
Pearson correlation	− 0.05	− 0.117	− 0.163
*p*-value (2-tailed)	0.693	0.412	0.253
*N*	66	51	51
Absolute error of saccade gain			
Control			
Pearson correlation	0.121	− 0.011	0.392*
*p*-value (2-tailed)	0.429	0.936	0.003
*N*	45	55	55
ASD			
Pearson correlation	− 0.064	− 0.094	0.169
*p*-value (2-tailed)	0.651	0.49	0.213
*N*	52	56	56

^*^significant based on adjusted FDR of *p* < .015

#### Reaction time—feedback-guided motor adjustments

Force reaction time was associated with increased CoV (15%, 45% MVC) in participants with ASD (Table 3). Saccade latency was associated with increased CoV (21.13 degrees) in control participants, and the strength of this relationship was stronger in controls than in ASD (*z* = 2.00, *p* = 0.045; Additional file 4: Fig. S3). Variability of saccade latency was not associated with CoV. Force reaction time, saccade latency, and trial-wise variability of saccade latency were not associated with variability of saccade error.

**Table 3:** Correlations between reaction time and feedback-guided motor adjustments

	Primary pulse reaction time	Saccade latency	Latency variability
Coefficient of variation—15% MVC			
Control			
Pearson correlation	0.18	0.3	0.2
*p*-value (2-tailed)	0.184	0.026	0.142
*N*	56	55	55
ASD			
Pearson correlation	0.345*	0.189	0.127
*p*-value (2-tailed)	0.004	0.163	0.349
*N*	68	56	56
Coefficient of variation—45% MVC			
Control			
Pearson correlation	0.203	0.327	0.285
*p*-value (2-tailed)	0.149	0.017	0.039
*N*	52	53	53
ASD			
Pearson correlation	0.348*	− 0.01	0.067
*p*-value (2-tailed)	0.005	0.943	0.625
*N*	65	55	55
Coefficient of variation—85% MVC			
Control			
Pearson correlation	0.125	0.218	0.095
*p*-value (2-tailed)	0.381	0.121	0.501
*N*	51	52	52
ASD			
Pearson correlation	− 0.049	0.067	0.214
*p*-value (2-tailed)	0.704	0.637	0.127
*N*	63	52	52
Coefficient of variation—0.06 degrees			
Control			
Pearson correlation	− 0.049	0.187	0.144
*p*-value (2-tailed)	0.763	0.254	0.38
*N*	41	39	39
ASD			
Pearson correlation	0.253	0.181	0.043
*p*-value (2-tailed)	0.163	0.32	0.817
*N*	32	32	32
Coefficient of variation—0.62 degrees			
Control			
Pearson correlation	− 0.083	0.359	0.143
*p*-value (2-tailed)	0.6	0.023	0.38
*N*	42	40	40
ASD			
Pearson correlation	0.259	0.114	0.241
*p*-value (2-tailed)	0.122	0.52	0.17
*N*	37	34	34
Coefficient of variation—21.13 degrees			
Control			
Pearson correlation	0.13	0.414*†	0.19
*p*-value (2-tailed)	0.41	0.008	0.24
*N*	42	40	40
ASD			
Pearson correlation	0.208	− 0.046	− 0.068
*p*-value (2-tailed)	0.245	0.808	0.723
*N*	33	30	30
Saccade gain variability			
Control			
Pearson correlation	0.216	− 0.046	0.255
* p*-value (2-tailed)	0.155	0.74	0.06
* N*	45	55	55
ASD			
Pearson correlation	− 0.074	0.057	0.182
* p*-value (2-tailed)	0.605	0.674	0.18
*N*	51	56	56

^*^Significant based on adjusted FDR of *p* < .01; †correlation significantly differs between groups

#### Initial action output—feedback-guided motor adjustments

Primary pulse accuracy was not associated with CoV (Table 4). In participants with ASD, increased saccade error was associated with increased CoV (85% MVC). Primary pulse error was not associated with saccade error. Saccade error was associated increased trial-to-trial variability of saccade error in both ASD and controls.

**Table 4:** Correlations between initial action output and feedback-guided motor adjustments

	Primary pulse absolute error	Absolute error of saccade gain
Coefficient of variation—15% MVC		
Control		
Pearson correlation	− 0.315	0.08
* p*-value (2-tailed)	0.018	0.561
N	56	55
ASD		
Pearson correlation	0.128	0.06
* p*-value (2-tailed)	0.307	0.655
*N*	66	57
Coefficient of variation—45% MVC		
Control		
Pearson correlation	− 0.141	0.244
*p*-value (2-tailed)	0.32	0.078
*N*	52	53
ASD		
Pearson correlation	− 0.013	0.297
*p*-value (2-tailed)	0.917	0.026
*N*	63	56
Coefficient of variation—85% MVC		
Control		
Pearson correlation	− 0.066	0.139
*p*-value (2-tailed)	0.648	0.327
*N*	51	52
ASD		
Pearson correlation	0.045	0.357*
* p*-value (2-tailed)	0.731	0.009
*N*	61	53
Coefficient of variation—0.06 degrees		
Control		
Pearson correlation	− 0.16	0.028
* p*-value (2-tailed)	0.317	0.867
*N*	41	39
ASD		
Pearson correlation	− 0.191	0.226
*p*-value (2-tailed)	0.295	0.213
*N*	32	32
Coefficient of variation—0.62 degrees		
Control		
Pearson correlation	− 0.312	0.004
*p*-value (2-tailed)	0.044	0.981
*N*	42	40
ASD		
Pearson correlation	− 0.246	0.301
*p*-value (2-tailed)	0.148	0.084
*N*	36	34
Coefficient of variation—21.13 degrees		
Control		
Pearson correlation	− 0.17	− 0.021
*p*-value (2-tailed)	0.281	0.9
*N*	42	40
ASD		
Pearson correlation	− 0.368	0.045
*p*-value (2-tailed)	0.038	0.812
*N*	32	30
Saccade gain variability		
Control		
Pearson correlation	0.301	.676*
*p*-value (2-tailed)	0.045	< .001
N	45	55
ASD		
Pearson correlation	− 0.039	.614*
*p*-value (2-tailed)	0.788	< .001
*N*	51	56

*Significant based on adjusted FDR of *p* < .01

#### Reaction time—time-dependent structure

Force reaction time was associated with reduced ApEn (15%, 45%, 85% MVC) in both ASD and controls (Table 5), although the association did not survive corrections for multiple comparisons for ASD at 85% MVC. Saccade latency was not associated with ApEn. In control participants, increased latency variability was associated with reduced ApEn (45% MVC).

**Table 5:** Correlations between reaction time and the time-dependent force structure

	Primary pulse reaction time	Saccade latency	Latency variability
Approximate entropy—15% MVC			
Control			
Pearson correlation	− 0.438*	− 0.202	− 0.218
*p*-value (2-tailed)	0.001	0.14	0.11
*N*	56	55	55
ASD			
Pearson correlation	− 0.448*	− 0.125	− 0.167
*p*-value (2-tailed)	< .001	0.359	0.22
*N*	68	56	56
Approximate entropy—45% MVC			
Control			
Pearson correlation	− 0.465*	− 0.283	− 0.474*
*p*-value (2-tailed)	0.001	0.04	< .001
*N*	52	53	53
ASD			
Pearson correlation	− 0.397*	− 0.19	− 0.174
*p*-value (2-tailed)	0.001	0.165	0.203
*N*	65	55	55
Approximate entropy—85% MVC			
Control			
Pearson correlation	− 0.470*	− 0.243	− 0.262
*p*-value (2-tailed)	0.001	0.083	0.061
*N*	51	52	52
ASD			
Pearson correlation	− 0.305	− 0.133	− 0.151
*p*-value (2-tailed)	0.015	0.348	0.284
*N*	63	52	52
Approximate ENTROPY—0.06 degrees			
Control			
Pearson correlation	− 0.292	− 0.204	− 0.015
*p*-value (2-tailed)	0.064	0.214	0.93
*N*	41	39	39
ASD			
Pearson correlation	− 0.199	− 0.155	− 0.316
*p*-value (2-tailed)	0.276	0.396	0.078
*N*	32	32	32
Approximate entropy—0.62 degrees			
Control			
Pearson correlation	− 0.223	− 0.225	− 0.02
*p*-value (2-tailed)	0.155	0.162	0.905
*N*	42	40	40
ASD			
Pearson correlation	− 0.143	− 0.097	− 0.25
*p*-value (2-tailed)	0.398	0.584	0.154
*N*	37	34	34
Approximate entropy—21.13 degrees			
Control			
Pearson correlation	− 0.224	− 0.286	− 0.084
*p*-value (2-tailed)	0.153	0.073	0.604
*N*	42	40	40
ASD			
Pearson correlation	− 0.21	0.02	0.15
*p*-value (2-tailed)	0.241	0.916	0.429
*N*	33	30	30

*significant based on adjusted FDR of p < *.01*

#### Initial action output—time-dependent structure

Primary pulse error was not associated with ApEn (Table 6). In control participants, increased saccade error was associated with reduced ApEn (15, 45, 85% MVC) and the strength of this association was stronger in controls than in ASD (45%; *z* = − 2.43, *p* = 0.015; Additional file 4: Fig. S4).

**Table 6:** Correlations between initial action output and the time-dependent force structure

	Primary pulse absolute error	Absolute error of saccade gain
Approximate entropy—15% MVC		
Control		
Pearson correlation	0.034	− 0.414*
*p*-value (2-tailed)	0.805	0.002
*N*	56	55
ASD		
Pearson correlation	− 0.159	− 0.123
*p*-value (2-tailed)	0.203	0.36
*N*	66	57
Approximate entropy—45% MVC		
Control		
Pearson correlation	0.14	− .526*†
*p*-value (2-tailed)	0.324	< .001
*N*	52	53
ASD		
Pearson correlation	0.047	− 0.105
*p*-value (2-tailed)	0.717	0.441
*N*	63	56
Approximate entropy—85% MVC		
Control		
Pearson correlation	− 0.123	− .378*
*p*-value (2-tailed)	0.39	0.006
*N*	51	52
ASD		
Pearson correlation	− 0.027	− 0.25
*p*-value (2-tailed)	0.838	0.071
*N*	61	53
Approximate entropy—0.06 degrees		
Control		
Pearson correlation	0.141	− 0.275
*p*-value (2-tailed)	0.38	0.09
*N*	41	39
ASD		
Pearson correlation	0.361	− 0.343
*p*-value (2-tailed)	0.042	0.055
*N*	32	32
Approximate entropy—0.62 degrees		
Control		
Pearson correlation	0.337	− 0.289
*p*-value (2-tailed)	0.029	0.071
*N*	42	40
ASD		
Pearson correlation	0.288	− 0.207
*p*-value (2-tailed)	0.088	0.24
*N*	36	34
Approximate entropy—21.13 degrees		
Control		
Pearson correlation	0.095	− 0.225
*p*-value (2-tailed)	0.549	0.163
*N*	42	40
ASD		
Pearson correlation	0.227	0.04
*p*-value (2-tailed)	0.212	0.834
N	32	30

^*^significant based on adjusted FDR of *p* < .01; †Correlation significantly differs between groups

#### Feedback-guided motor adjustments—time-dependent structure

Within each task condition, increased CoV was associated with reduced ApEn (Table 7) in both ASD and controls. In control participants, increased variability of saccade gain was associated with reduced ApEn (15, 45, 85% MVC).

**Table 7:** Correlations between feedback-guided motor adjustments and the time-dependent force structure

	Coefficient of variation—15% MVC	Coefficient of variation—45% MVC	Coefficient of variation—85% MVC	Coefficient of variation—0.06 degrees	Coefficient of variation—0.62 degrees	Coefficient of variation—21.13 degrees	Saccade gain variability
Approximate entropy—15% MVC							
Control							
Pearson correlation	− 0.418*	− 0.572*	− 0.420*	− 0.265	− 0.363*	− 0.398*	− 0.412*
*p*-value (2-tailed)	< .001	< .001	< .001	0.057	0.006	0.003	0.002
*N*	72	68	66	52	55	54	55
ASD							
Pearson correlation	− 0.652*	− 0.615*	− 0.396*	− 0.673*	− 0.529*	− 0.332*	− 0.272
*p*-value (2-tailed)	< .001	< .001	0.001	< .001	< .001	0.048	0.043
*N*	75	71	69	37	42	36	56
Approximate entropy—45% MVC							
Control							
Pearson correlation	− 0.416*	− 0.710*	− 0.518*	− 0.538*	− 0.468*	− 0.501*	− 0.479*
*p*-value (2-tailed)	< .001	< .001	< .001	< .001	0.001	< .001	< .001
*N*	68	68	66	48	51	50	53
ASD							
Pearson correlation	− 0.473*	− 0.715*	− 0.594*	− 0.519*	− 0.350	− 0.568*	− 0.307
*p*-value (2-tailed)	< .001	< .001	< .001	0.001	0.025	< .001	0.022
*N*	71	71	68	36	41	35	55
Approximate entropy—85% MVC							
Control							
Pearson correlation	− 0.342*	− 0.553*	− 0.669*	− 0.380*	− 0.344	− 0.319	− 0.412*
*p*-value (2-tailed)	0.005	< .001	< .001	0.009	0.015	0.027	0.002
*N*	66	66	66	46	49	48	52
ASD							
Pearson correlation	− 0.285*	− 0.471*	− 0.516*	− 0.365	− 0.139	− 0.27	− 0.258
*p*-value (2-tailed)	0.018	< .001	< .001	0.037	0.405	0.135	0.064
*N*	69	68	69	33	38	32	52
Approximate entropy—0.06 degrees							
Control							
Pearson correlation	− 0.475*	− 0.583*	− 0.562*	− 0.535*	− 0.451*	− 0.381*	− 0.165
*p*-value (2-tailed)	< .001	< .001	< .001	< .001	0.001	0.006	0.316
N	52	48	46	52	52	51	39
ASD							
Pearson correlation	− 0.585*	− 0.487*	− 0.586*	− 0.645*	− 0.519*	− 0.359*	− 0.048
*p*-value (2-tailed)	< .001	0.003	< .001	< .001	0.001	0.047	0.795
*N*	37	36	33	37	37	31	32
Approximate entropy—0.62 degrees							
Control							
Pearson correlation	− 0.540*	− 0.618*	− 0.509*	− 0.496*	− 0.613*	− 0.482*	− 0.137
*p*-value (2-tailed)	< .001	< .001	< .001	< .001	< .001	< .001	0.399
*N*	55	51	49	52	55	54	40
ASD							
Pearson correlation	− 0.735*	− 0.524*	− 0.466*	− 0.666*	− 0.746*	− 0.510*	− 0.25
*p*-value (2-tailed)	< .001	< .001	0.003	< .001	< .001	0.001	0.154
*N*	42	41	38	37	42	36	34
Approximate entropy—21.13 degrees							
Control							
Pearson correlation	− 0.419*	− 0.548*	− 0.282	− 0.378*	− 0.412*	− 0.670*	− 0.074
*p*-value (2-tailed)	0.002	< .001	0.052	0.006	0.002	< .001	0.652
*N*	54	50	48	51	54	54	40
ASD							
Pearson correlation	− 0.407*	− 0.754*	− 0.488*	− 0.436	− 0.292	− 0.800*	− 0.039
*p*-value (2-tailed)	0.014	< .001	0.005	0.014	0.084	< .001	0.84
*N*	36	35	32	31	36	36	30

*Significant based on adjusted FDR of *p* < .01

### Clinical correlations

Among individuals with ASD, severity of social interactions was associated with reduced saccade variability (*r* = − 0.34, *p* = 0.001). More severe communication abnormalities (ADI-R) were associated with increased CoV at high force (*r* = 0.30, *p* = 0.03), increased saccade dysmetria (*r* = 0.30, *p* = 0.009), and reduced saccade variability (*r* = − 0.25, *p* = 0.02). More severe clinically-rated RRBs (ADI-R) also were associated with increased saccade dysmetria (*r* = 0.33, *p* = 0.003).

## Discussion

We examined sensorimotor behavior across different effector systems (hand, eye) to characterize initial motor output (saccade gain, primary pulse accuracy), feedback-guided motor processes (saccade gain variability, force variability), and their time dependent structure (entropy) across a relatively large sample of children, adolescents and adults with ASD. Three key findings are highlighted. First, we present new results indicating initial action output and feedback-guided motor adjustments each are compromised in ASD, but the extent to which these behaviors are impacted varies as a function of the rate at which they are executed. Specifically, increased variability of precision grip force and reduced accuracy of saccades in ASD relative to controls implicates reduced precision of rapid motor output. In contrast, slower occurring adjustments of initial grip force output and saccade amplitude consistency across separate trials are relatively unaffected in ASD suggesting that increased time for modulating motor output may contribute to partial recovery of motor precision. Second, consistent with prior studies, individuals with ASD showed reduced lateralization of grip strength suggesting atypical hemispheric specialization of motor systems [11, 53, 54]. Third, sensorimotor issues varied with severity of social-communication and RRB symptoms suggesting sensorimotor dysfunctions may play a central role in the development of core features of ASD.

### Initial action output is affected for eye movements but not precision grip force in ASD

We examined initial motor output by measuring the accuracy of visually-guided saccades and initial force output during precision gripping. Similar to previous studies, we find evidence for reduced saccade accuracy in ASD [38, 61, 66], implicating reduced accuracy of forward control processes that execute internal action models to support rapid, ballistic movements. Internal models are formed as a result of repeated engagement in behavior, refined via corrective error signals, and support precise motor control of actions that are executed too rapidly to be modulated by slower feedback processes [24]. Our findings of reduced saccade accuracy in ASD, in the absence of increased trial-to-trial saccade variability, suggest these deficits may specifically be related to reduced precision of the internal model rather than increased noise of visual feedback that would contribute to reduced ability to modulate saccade amplitude precision across time or trials. Reduced stability of the internal model is consistent with recent findings showing heightened encoding of sensory prediction errors in ASD suggesting environmental unpredictability is overestimated in ASD [32]. Additionally, we find that saccade dysmetria in ASD primarily involves hypometric saccades. In light of recent data demonstrating reduced visual cortical representation of peripheral space in ASD [15], these findings may reflect deficient sensory processing of lateral (relative to central) visual targets that contribute to reduced amplitude of precision eye movements. This hypothesis is consistent with prior studies showing that saccade dysmetria in ASD is more severe at larger target step amplitudes [44, 61].

Increased age was associated with greater saccade accuracy in controls but not in ASD suggesting attenuation of maturational processes that support refinement of feedforward motor control processes into early neurotypical adulthood [39]. Our results contrast with those of previous studies that have demonstrated reduced saccade accuracy that is more severe in children compared to adults [38]. As we also find that saccade dysmetria covaries with the severity of core ASD symptoms, it is possible that differences between our results and those of Luna et al. may reflect distinctions in the severity of individuals studied. This possibility is further emphasized by findings that saccade dysmetria may be more pronounced in or specific to individuals with ASD and history of language delay [66]. Differences in age-associations between our study and Luna et al. [38] also may reflect differences in target step amplitudes (Luna et al. tested targets at ± 10, 20, and 30 degrees) as individuals with ASD appear to show more severe dysmetria at higher amplitudes [61]. Longitudinal studies of saccade accuracy across a range of severity of core and associated symptoms and across multiple target step amplitudes are needed to clarify patterns of maturation of feedforward motor control processes supporting oculomotor behavior.

Examining initial force output during precision gripping, we did not identify primary pulse accuracy differences between individuals with ASD and controls, contrary to our hypotheses. Relative to rapid visually-guided saccades, initial grip force increases over an extended time scale suggesting output is guided both by rapid, feedforward control processes and feedback modulation involving haptic, somatosensory, and visual input. Our findings of reduced saccade accuracy but intact initial force output suggest that alterations in forward control models in ASD may be somewhat mitigated by slower sensory feedback processes to increase precision of motor output executed in the periphery. Feedback contributions to initial motor control highlight important differences in the neurophysiology of these effector systems, including increased utilization of proprioceptive input in skeletomotor relative to oculomotor systems [1] as well as the contribution of haptic feedback to precision manual motor control. In line with these findings, increased reliance on feedback-driven control processes has been documented in young children with ASD [7, 16, 17] and may be particularly driven by dependence on proprioceptive cues relative to other feedback modalities, as suggested previously [19, 26]. Finally, we document no association between saccade accuracy and primary pulse accuracy in participants with ASD or controls, suggesting that while both visually-guided saccades and initial grip force output involve relatively rapid motor behaviors, these measures capture distinct aspects of sensorimotor control.

Our findings of intact primary pulse accuracy in ASD differ from our previous studies of initial force output in ASD that have identified reduced accuracy (target overshoot) at very low force (e.g., 5% MVC; [47]) and at 15% MVC when individuals utilize a specific type of primary pulse characterized by an initial rapid increase followed by rapid release in force (Type 1), often resulting in a transient overshoot of the target [75]. We chose not to decompose separate pulse types in the present study because we previously found that Type 1 responses become decreasingly common during trials of longer intervals suggesting that they reflect a motor plan involving rapid exertion that is not well-suited for actions of longer duration or greater effort (i.e. increased MVC; [74]). Our current findings thus suggest that paradigms with extended trial duration (e.g., 15 s) may involve execution of initial motor plans that involve more gradual increases in force, such that initial output does not exceed task demands (target force level) and can be refined by feedback inputs, limiting deficits in feedforward control to manifest in individuals with ASD. This hypothesis is consistent with our prior results showing that individuals with ASD show similar levels of primary pulse accuracy as controls at 15% MVC when multiple different pulse types are averaged, as we did here [74].

### Feedback modulation of motor output is disrupted during grip force but not eye movements in ASD

We examined feedback-guided motor adjustments by measuring rapid modulation of continuous force output (CoV) and trial-to-trial variability of saccade gain. Consistent with prior studies, we found that individuals with ASD show greater grip force variability than controls [47, 72, 74]. Several separate motor control mechanisms could underpin this deficit in ASD. Based on the present findings, we propose that greater force variability reflects a reduced ability to rapidly integrate multi-sensory feedback and feedforward control processes to precisely adjust ongoing motor behavior. This hypothesis is supported by multiple lines of evidence. First, we find that increased force variability is highly associated with reduced force entropy in ASD (Table 7) implicating deficient ability to integrate the multiple motor control processes (including different sensory feedback inputs as well as feedforward controllers) that operate on different time-scales to dynamically adjust ongoing force output. These findings are consistent with our prior study showing increased reliance on slower feedback processes during sustained grip force in ASD [47]. Second, increased force variability also was associated with increased force reaction time in ASD suggesting that a reduced ability to rapidly execute manual motor commands contributes to deficits in precise modulation of sustained output. These results build on previous findings of deficient temporal coordination of manual motor control in ASD that implicate disruptions in processes for effective deployment of anticipatory, feedforward motor plans [6, 7]. In the current study, we document similar overall reaction times in ASD and controls suggesting that it is not the ability to rapidly initiate motor behavior, but the abilities to rapidly process multisensory feedback and translate sensory error into a precise motor command that are compromised in ASD. Third, force variability was elevated in ASD across all force and gain levels tested, but these elevations were more severe and consistent across ages during conditions in which visual spatial error signals were amplified (highest force level and highest gain) and the demand to rapidly process and translate feedback was greatest. As a result, the impact of temporal lag on motor adjustments is more severe during high force and high gain conditions. These findings are consistent with our prior study demonstrating elevations in 0–4 Hz power and reductions in 4–12 Hz power in individuals with ASD relative to controls, especially during conditions of high force and high gain, implicating deficient ability to rapidly and dynamically adapt ongoing motor behavior in response to error information [47].

Multiple additional sensorimotor control processes also could contribute to elevated force variability in ASD. It is possible that elevated force variability reflects increases in intrinsic noise within the motor system and a reduced ability to consistently attenuate this noise. One prior study demonstrated increased variability of beta and gamma frequency and decreased delta frequency motorneuron firing during single finger abduction (first dorsal interosseus) in ASD relative to controls suggesting atypical modulation of motorneuron pool activity during sustained manual force [73]. Interestingly, motorneuron pool activity (delta, beta and gamma) was highly associated with force variability only in controls but not individuals with ASD suggesting reduced synergy between motorneuron firing and force output variability in ASD. Analyses of relationships between intrinsic motorneuron activity and motor variability in ASD will be important for testing the hypothesis that intrinsic variation may contribute to deficits modulating motor output.

We find that elevations in force variability are more severe in young children with ASD and show more rapid age-associated improvements relative to controls at lower levels of force and reduced visual gain, suggesting that the ability to rapidly adjust precision motor output may be mitigated over time under certain conditions, such as those that are less demanding on the motor system (e.g., low force) or for which visual feedback is presented less rapidly (low gain relative to high gain). Early issues in sensory feedback control of motor behavior may reflect atypical sensory and multisensory processing as suggested by prospective studies demonstrating rapid over-expansion of sensory cortex in infants who are later diagnosed with ASD [20] that could contribute to persistent deficits in rapid integration of sensory feedback. Importantly, these results also suggest that measurement of precision gripping variability at low force levels may help reliably differentiate children with ASD from age-matched TD children in line with recent data demonstrating that early emerging impairments in fine motor, but not gross motor, abilities were predictive of later ASD severity [25]. Our results also converge with recent studies demonstrating the importance of characterizing spatial and temporal aspects of visual-motor integration for characterizing heterogeneity across ASD symptom profiles and differentiating individuals with ASD from other neurodevelopmental disorders [35, 70]. Deficits in early motor development in ASD appear to be predictive of familial recurrence suggesting that tracking select motor behaviors may provide important insights into both early emerging neurodevelopmental mechanisms and important targets for monitoring early emerging ASD risk factors [21, 34, 57].

In contrast to deficits in rapid adjustment of motor behavior and contrary to our hypotheses, we do not document differences in ASD relative to controls in variability of saccade amplitudes across trials. In the case of eye movements, sensory feedback is used to modulate the amplitude or duration of the subsequent saccade to minimize endpoint error [50, 55]. Our findings that trial-to-trial saccade amplitude variability in ASD was only modestly associated with grip force variability (*R*^2^ = 0.12-0.16) suggests that feedback guided modulation of saccade and grip force output involves only partially overlapping but also distinct mechanisms. We propose that distinctions between the two processes reflect separate time scales that include rapid adjustments to grip force during sustained contractions in contrast with the more protracted time course of updates to internal action representations afforded by inter-stimulus intervals (1.5–2.5 s) of our visually guided saccade test.

Our findings of overall saccade dysmetria suggest reduced precision of the internal model in ASD that may arise from deficiencies in consolidation of sensory feedback, while our findings of intact saccade variability suggest that error-reducing processes that occur over a protracted course may be relatively spared in ASD. However, previous studies have documented greater trial-to-trial saccade variability [61, 66] and reduced adaptation of saccade amplitudes following experimentally induced error [46]. Differences in findings between studies may be attributable to the use of only one target step amplitude relative to three as in Schmitt et al. [61]. However, our data demonstrating that variability of saccade gain was more pronounced in individuals with ASD with less severe impairments in reciprocal social interaction and communication abnormalities converges with findings from Takarae et al. [66] showing more severe elevations in trial-wise saccade amplitude variability in individuals with ASD without language delays relative to those with delayed language. These data suggest our findings regarding saccade variability in ASD may reflect differences in sample characteristics, including the use of younger participants and individuals with reduced symptom severity, and in the nature of the paradigm that minimized variation due to variable locations of peripheral stimuli.

### Deficits in time-dependent structure reflect greater reliance on slower feedback mechanisms in ASD

Dynamic adaptation of behavior is the product of integrated inputs across multiple modalities that operate on different timescales, including rapid feedforward processes and slower visual, proprioceptive, and haptic feedback processes. The integration of these multiple control processes is reflected in the time-dependent structure of the motor output, with greater irregularity (ApEn) reflecting greater integration of distinct motor control processes (e.g., [56]). Consistent with our previous studies, we found reduced ApEn in ASD relative to controls suggesting a more rigid and less dynamic sensorimotor control strategy [47]. Importantly, reductions in ApEn were more pronounced in ASD relative to controls at high levels of force, when using the non-dominant hand, and when visual feedback error information was amplified (high gain). These findings suggest that when motor behaviors are more difficult (e.g., at higher force levels, with non-dominant hand), individuals with ASD show a more severe deterioration than controls in their ability to dynamically adjust output due to greater regularity of force oscillations, or decreased ability to leverage multiple motor control processes. The hypothesis that individuals with ASD may become less able to integrate multi-sensory inputs and increasingly reliant on discrete primary or dominant mechanisms such as internal action plans or visual feedback during more challenging motor tasks is consistent with prior studies showing greater weighting of single modality sensory inputs during motor learning [19, 47] and inflexibility in weighting sensory prediction error across contexts [51]. Findings that ApEn is reduced to a greater degree in ASD relative to controls at high compared to low and medium gains suggests that individuals with ASD are able to increase the dynamism of their force control in response to changes in sensory feedback, but that this ability is attenuated, perhaps reflecting a relative ceiling on their capacity to integrate multisensory information and forward models.

### Lateralization of grip strength is reduced in ASD

We document reductions in grip strength (MVC) in ASD relative to controls across both dominant and non-dominant hands, but these contrasts were more severe in the dominant hand suggesting reduced lateralization of gross motor strength. These findings converge with previous studies demonstrating increased rates of mixed-handedness and reduced differentiation of dominant relative to non-dominant limb control in ASD [11, 53, 54]. Importantly, our findings of reduced lateralization of grip strength in ASD also are similar to a prior evaluation of neuromuscular tone showing that 61% of individuals with ASD exhibit no tonic laterality of the upper limbs [52]. Our results also are consistent with quantitative measures of brain laterality, including a functional magnetic resonance imaging (fMRI) study documenting associations between atypical (increased ipsilateral cortex) lateralization of sensorimotor network connectivity in ASD and more severe clinical neuromotor difficulties [12]. Collectively, these findings implicate reduced hemispheric specialization of key sensorimotor networks that also may impact the development of other key cognitive and behavioral abilities associated with ASD (e.g., language; [13]).

### Core symptoms of ASD are associated with severity of initial actions and feedback-modulated motor output

We found that greater saccade error and force variability in ASD each were related to more severe clinically-rated communication abnormalities suggesting overlapping neurodevelopmental processes. Together these associations indicate that deficits in motor behaviors occurring on a rapid timescale may contribute to developmental disruptions in rapid processing and coordinated response timing for dynamic social and communication inputs as suggested previously [23, 43, 58]. Our results also show that more severe saccade dysmetria is associated with increased severity of RRBs in ASD. It is possible that this correlation merely represents the broad contribution of sensorimotor alterations on neurodevelopmental disruptions that impact core clinical features. Alternatively, this finding could reflect a more specific association implicated by preclinical models and clinical fMRI and structural MRI studies implicating frontostriatal abnormalities in both saccade control and the pathophysiology of RRBs [33, 62, 67]. Finally, associations between reduced trial-wise variability of saccade accuracy and more severe communication deficits in ASD suggest select sensorimotor behaviors may show developmental divergence with core ASD symptoms. This finding builds on recent work positing that sensorimotor and autism-associated traits represent distinct endophenotypes associated with separate etiologic pathways of ASD [5, 49] and is consistent with a prior eye movement study showing that language delay is associated with *less* severe elevations in trial-wise saccade amplitude variability [66]. Collectively, our results suggest that sensorimotor issues in ASD show both concurrence and divergence with core symptoms. These findings indicate that these separate features may show overlapping pathophysiology, but that sensorimotor function should be considered in as a multidimensional construct with variable expression of select behaviors across the autism spectrum.

### Limitations and implications for future research

While we provide new results parsing distinct sensorimotor behaviors and their impact in ASD, our results should be considered in the context of multiple limitations. A primary limitation of this study is the reliance on cross-sectional rather than longitudinal data. While our large sample spanning a wide age-range provides important landmarks for tracking development in multiple sensorimotor control processes, this data also underscores the heterogeneity of sensorimotor issues across motor systems and individuals. Specifically, cross-sectional data does not allow for the parsing of inter-individual heterogeneity which has the potential to confound age-related effects identified in this study. Additionally, our sample is limited by unequal distributions of participants across ages including fewer participants over age 15 years which may have led to less robust estimations of age-related changes in older adolescent and adult participants. Therefore, indications of disrupted maturational processes in ASD should be interpreted with caution until these findings are replicated in longitudinal cohorts. A second limitation of our study is that the majority of our participants with ASD had average to above average IQs; therefore, these results may not be generalizable to individuals with ASD and comorbid intellectual/developmental disability. While we did not see strong associations between cognitive ability and motor behavior in our sample, it is possible that sensorimotor issues may covary more strongly when studying individuals outside of the normal range of IQ as suggested previously [68, 69]. Finally, a number of our conclusions are predicated on relatively small effect sizes, particularly for oculomotor variables and cross-task associations, and we emphasize the need for replication with larger samples and longitudinal studies of within-participant variation across these measures over time.

## Conclusions

This study is among the first to characterize initial action output and feedback-guided motor behavioral precision in ASD across effector systems and a wide range of development. Our data indicate that feedforward and feedback processes are disrupted in ASD and that the degree to which these disruptions interact to result in atypical sensorimotor behavior varies across effector and demand on the motor system. We also document reduced lateralization of grip strength in ASD implicating atypical hemispheric specialization. These results highlight the strong need to parse separate sensorimotor behaviors and track them longitudinally to define spared and affected systems in ASD and track their change during development. Together with prior studies showing that motor deficits are common and early emerging in ASD, our findings of impairments in precision motor behavior in young children with ASD that covary with core communication symptoms highlight the potential utility of tracking select sensorimotor behaviors to promote earlier and more objective identification strategies.

## Supplementary Information

**Additional file 1**. Age distributions by diagnostic group.

**Additional file 2**. Trial exclusion by task, group, and age.

**Additional file 3**. Linear mixed effects model results.

**Additional file 4**. Additional figures 1–4.

## Data Availability

The datasets used and/or analyzed during the current study are available from the corresponding author on reasonable request.

## Abbreviations

ASD: Autism Spectrum Disorder
TD: Typically developing
UIC: University of Illinois at Chicago
UTSW: University of Texas Southwestern Medical Center
DAS: Differential Ability Scale
WASI: Wechsler Abbreviated Scale of Intelligence
WPPSI: Wechsler Preschool and Primary Scales of Intelligence
ADI-R: Autism Diagnostic Inventory-Revised
ADOS: Autism Diagnostic Observation Schedule—Second Edition
DSM: Diagnostic and Statistical Manual of Mental Disorders
MVC: Maximum voluntary contraction
CoV: Coefficient of variation
ApEn: Approximate entropy
SD: Standard deviation
CSS: Calibrated severity score
RBS-R: Repetitive Behavior Scale-Revised
VGS: Visually-guided saccade
