# Supplementary figures and images for: Initial action output and feedback-guided motor behaviors in autism spectrum disorder
Source: Mol Autism. 2021 Jul 10;12:52. doi: 10.1186/s13229-021-00452-8 (PMC8272343; doi:10.1186/s13229-021-00452-8)

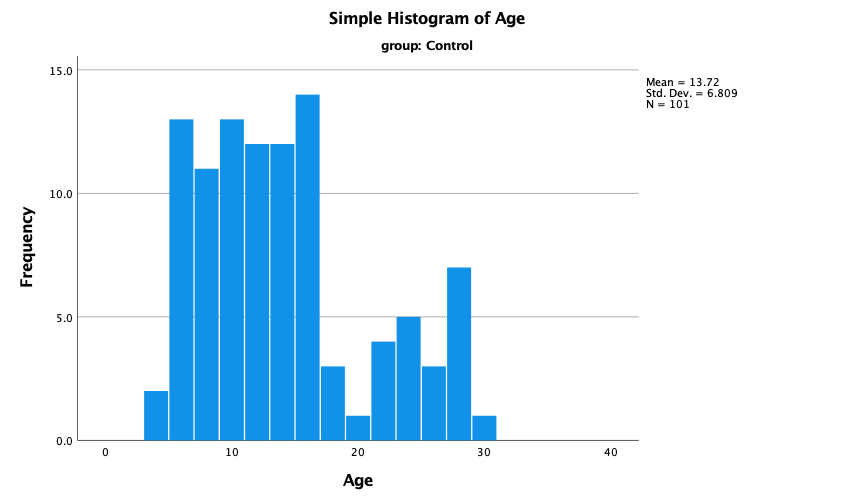


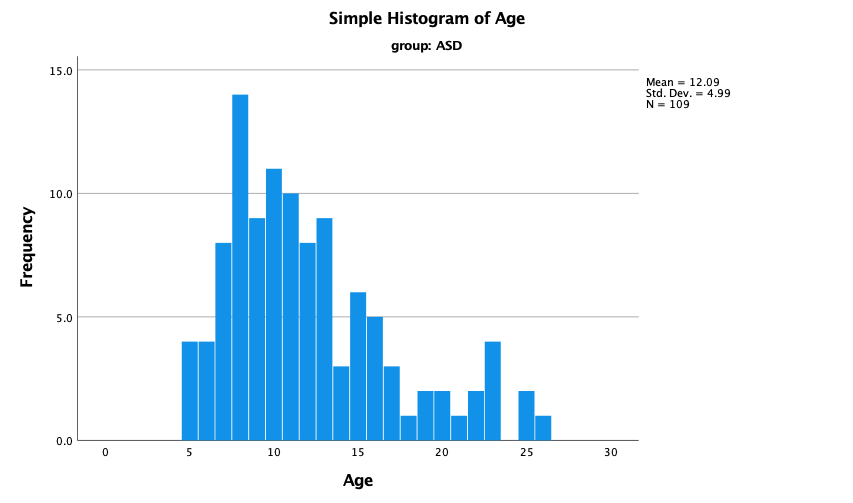

Supplement: Supplementary file 1 — Additional file 1. Age distributions by diagnostic group. [file 13229_2021_452_MOESM1_ESM.docx]
