# Supplementary material for: Initial action output and feedback-guided motor behaviors in autism spectrum disorder
Source: Mol Autism. 2021 Jul 10;12:52. doi: 10.1186/s13229-021-00452-8 (PMC8272343; doi:10.1186/s13229-021-00452-8)
Supplement: Supplementary file 2 — Additional file 2. Trial exclusion by task, group, and age. [file 13229_2021_452_MOESM2_ESM.docx]

**Models including sex as a covariate:**

Signif. codes: 0 ‘***’ 0.001 ‘**’ 0.01 ‘*’ 0.05 ‘.’ 0.1 ‘ ’ 1

Maximum voluntary contraction

Type III Analysis of Variance Table with Satterthwaite's method

Sum Sq Mean Sq NumDF DenDF F value Pr(>F)

c_handtested 7518.5 7518.5 1 2103.59 188.0869 < 2.2e-16 ***

c_group 0.0 0.0 1 144.15 0.0010 0.97443

c_age 7024.9 7024.9 1 144.09 175.7404 < 2.2e-16 ***

c_location 3826.6 3826.6 1 144.09 95.7280 < 2.2e-16 ***

c_sex 203.2 203.2 1 144.27 5.0828 0.02567 *

c_handtested:c_group 2158.7 2158.7 1 2103.59 54.0035 2.849e-13 ***

c_handtested:c_age 34.5 34.5 1 2103.49 0.8637 0.35282

c_group:c_age 225.7 225.7 1 144.05 5.6471 0.01880 *

c_handtested:c_group:c_age 1235.8 1235.8 1 2103.48 30.9145 3.039e-08 ***

Primary pulse reaction time (No significant predictors identified)

Type III Analysis of Variance Table with Satterthwaite's method

Sum Sq Mean Sq NumDF DenDF F value Pr(>F)

c_sex 0.15551 0.15551 1 108.20 2.527 0.1148

c_location 1.08494 1.08494 1 108.95 17.629 5.503e-05 ***

Saccade latency (No significant predictors identified)

Type III Analysis of Variance Table with Satterthwaite's method

Sum Sq Mean Sq NumDF DenDF F value Pr(>F)

c_age 7238.8 7238.8 1 169 20.7888 9.806e-06 ***

c_location 328.3 328.3 1 169 0.9427 0.3330

c_sex 1.3 1.3 1 169 0.0037 0.9514

Variability of saccade latency

Type III Analysis of Variance Table with Satterthwaite's method

Sum Sq Mean Sq NumDF DenDF F value Pr(>F)

c_direction 10.94 10.94 1 167.91 0.0713 0.789809

c_group 491.83 491.83 1 166.21 3.2042 0.075270 .

c_age 1824.49 1824.49 1 166.59 11.8864 0.000716 ***

c_sex 12.64 12.64 1 166.28 0.0823 0.774511

c_location 530.96 530.96 1 166.54 3.4592 0.064666 .

c_direction:c_group 53.05 53.05 1 167.91 0.3456 0.557400

c_group:c_age 20.07 20.07 1 166.60 0.1307 0.718132

c_direction:c_age 217.62 217.62 1 168.22 1.4178 0.235446

c_direction:c_group:c_age 702.63 702.63 1 168.22 4.5775 0.033836 *

Primary pulse accuracy (No significant predictors identified)

Type III Analysis of Variance Table with Satterthwaite's method

Sum Sq Mean Sq NumDF DenDF F value Pr(>F)

c_sex 1.0800 1.0800 1 106.09 4.9729 0.02785 *

c_location 4.1047 4.1047 1 107.79 18.9012 3.132e-05 ***

Saccade gain

Type III Analysis of Variance Table with Satterthwaite's method

Sum Sq Mean Sq NumDF DenDF F value Pr(>F)

c_direction 0.073849 0.073849 1 4048.1 4.7257 0.02977 *

c_group 0.017116 0.017116 1 161.5 1.0953 0.29687

c_age 0.002570 0.002570 1 163.6 0.1645 0.68561

c_location 0.037451 0.037451 1 164.2 2.3966 0.12353

c_sex 0.002395 0.002395 1 161.9 0.1533 0.69595

c_group:c_age 0.104555 0.104555 1 164.3 6.6906 0.01056 *

Force test: Coefficient of variation

Type III Analysis of Variance Table with Satterthwaite's method

Sum Sq Mean Sq NumDF DenDF F value Pr(>F)

c_MVC.pct 2.71735 1.35868 2 1966.76 120.4167 < 2.2e-16 ***

c_group 0.11198 0.11198 1 140.91 9.9244 0.0019922 **

c_age 0.46478 0.46478 1 139.38 41.1928 2.011e-09 ***

c_location 0.13217 0.13217 1 137.61 11.7138 0.0008174 ***

c_sex 0.00880 0.00880 1 138.89 0.7798 0.3787423

c_MVC.pct:c_group 0.48302 0.24151 2 1967.88 21.4048 6.363e-10 ***

c_MVC.pct:c_age 0.02435 0.01217 2 1964.43 1.0789 0.3401763

c_group:c_age 0.02123 0.02123 1 139.35 1.8812 0.1724014

c_MVC.pct:c_group:c_age 0.14466 0.07233 2 1964.41 6.4104 0.0016790 **

Gain test: Coefficient of variation

Type III Analysis of Variance Table with Satterthwaite's method

Sum Sq Mean Sq NumDF DenDF F value Pr(>F)

c_visual.angle 1.38801 0.69401 2 1449.26 60.1623 < 2.2e-16 ***

c_group 0.29956 0.29956 1 90.76 25.9682 1.879e-06 ***

c_age 0.62518 0.62518 1 89.63 54.1958 8.408e-11 ***

c_location 0.00614 0.00614 1 91.12 0.5326 0.467380

c_sex 0.00991 0.00991 1 91.18 0.8592 0.356408

c_visual.angle:c_group 0.25157 0.12578 2 1452.19 10.9040 1.994e-05 ***

c_visual.angle:c_age 0.11867 0.05934 2 1449.27 5.1437 0.005943 **

c_group:c_age 0.08883 0.08883 1 90.37 7.7004 0.006709 **

c_visual.angle:c_group:c_age 0.12980 0.06490 2 1450.70 5.6261 0.003682 **

Saccade gain variability

Type III Analysis of Variance Table with Satterthwaite's method

Sum Sq Mean Sq NumDF DenDF F value Pr(>F)

c_age 0.013896 0.013896 1 166.25 14.7846 0.0001714 ***

c_location 0.049402 0.049402 1 166.35 52.5596 1.496e-11 ***

c_sex 0.000185 0.000185 1 167.42 0.1967 0.6579491

Force test: Approximate entropy

Type III Analysis of Variance Table with Satterthwaite's method

Sum Sq Mean Sq NumDF DenDF F value Pr(>F)

c_MVC.pct 2.82147 1.41073 2 1998.03 156.4035 < 2.2e-16 ***

c_group 0.08650 0.08650 1 135.94 9.5896 0.0023774 **

c_age 0.80933 0.80933 1 134.67 89.7273 < 2.2e-16 ***

c_location 1.09807 1.09807 1 132.87 121.7397 < 2.2e-16 ***

c_sex 0.00511 0.00511 1 134.42 0.5668 0.4528342

c_MVC.pct:c_group 0.09215 0.04608 2 1998.80 5.1084 0.0061252 **

c_MVC.pct:c_age 0.13364 0.06682 2 1994.65 7.4082 0.0006231 ***

c_group:c_age 0.00011 0.00011 1 134.46 0.0122 0.9123264

c_MVC.pct:c_group:c_age 0.11642 0.05821 2 1994.68 6.4536 0.0016079 **

Gain test: Approximate entropy

Type III Analysis of Variance Table with Satterthwaite's method

Sum Sq Mean Sq NumDF DenDF F value Pr(>F)

c_visual.angle 1.13054 0.56527 2 1400.11 75.0673 < 2.2e-16 ***

c_handtested 0.00300 0.00300 1 1407.59 0.3981 0.5281927

c_group 0.16333 0.16333 1 96.67 21.6906 1.022e-05 ***

c_age 0.28646 0.28646 1 95.45 38.0418 1.661e-08 ***

c_location 0.08851 0.08851 1 97.99 11.7547 0.0008890 ***

c_sex 0.00034 0.00034 1 97.07 0.0457 0.8311727

c_visual.angle:c_group 0.01465 0.00733 2 1404.08 0.9730 0.3782209

c_visual.angle:c_age 0.13408 0.06704 2 1399.43 8.9027 0.0001439 ***

c_group:c_age 0.00055 0.00055 1 96.14 0.0737 0.7866457

c_handtested:c_group 0.00000 0.00000 1 1408.00 0.0001 0.9935419

c_handtested:c_age 0.00069 0.00069 1 1404.57 0.0923 0.7613361

c_visual.angle:c_group:c_age 0.05252 0.02626 2 1401.81 3.4870 0.0308569 *

c_handtested:c_group:c_age 0.04568 0.04568 1 1404.79 6.0657 0.0139021 *

**Models including sex as a predictor:**

Maximum voluntary contraction

Type III Analysis of Variance Table with Satterthwaite's method

Sum Sq Mean Sq NumDF DenDF F value Pr(>F)

c_handtested 7130.9 7130.9 1 2106.76 177.1806 < 2.2e-16 ***

c_group 0.4 0.4 1 142.78 0.0101 0.919973

c_age 2410.1 2410.1 1 142.18 59.8848 1.698e-12 ***

c_location 3849.8 3849.8 1 142.13 95.6569 < 2.2e-16 ***

c_sex 345.3 345.3 1 142.62 8.5785 0.003961 **

c_age:c_sex 181.7 181.7 1 142.30 4.5144 0.035338 *

c_handtested:c_sex 69.4 69.4 1 2112.19 1.7252 0.189171

c_group:c_age:c_sex 210.4 105.2 2 142.43 2.6142 0.076735 .

c_handtested:c_group:c_sex 1702.0 851.0 2 2114.02 21.1450 8.081e-10 ***

Primary pulse reaction time (No significant predictors identified)

Type III Analysis of Variance Table with Satterthwaite's method

Sum Sq Mean Sq NumDF DenDF F value Pr(>F)

c_location 1.5321 1.5321 1 114.31 19.776 2.03e-05 ***

Saccade latency (No significant predictors identified)

Type III Analysis of Variance Table with Satterthwaite's method

Sum Sq Mean Sq NumDF DenDF F value Pr(>F)

c_direction 1.2 1.2 1 171 0.0034 0.9533890

c_group 4.4 4.4 1 166 0.0126 0.9108278

c_age 4458.4 4458.4 1 166 12.8816 0.0004365 ***

c_location 106.9 106.9 1 166 0.3089 0.5791006

c_sex 3.1 3.1 1 166 0.0089 0.9247516

c_age_sq 2511.8 2511.8 1 166 7.2572 0.0077871 **

c_direction:c_group 1054.4 1054.4 1 171 3.0463 0.0827168 .

c_group:c_sex 1512.2 1512.2 1 166 4.3690 0.0381223 *

Variability of saccade latency

Type III Analysis of Variance Table with Satterthwaite's method

Sum Sq Mean Sq NumDF DenDF F value Pr(>F)

c_direction 10.94 10.94 1 167.91 0.0713 0.789809

c_group 491.83 491.83 1 166.21 3.2042 0.075270 .

c_age 1824.49 1824.49 1 166.59 11.8864 0.000716 ***

c_sex 12.64 12.64 1 166.28 0.0823 0.774511

c_location 530.96 530.96 1 166.54 3.4592 0.064666 .

c_direction:c_group 53.05 53.05 1 167.91 0.3456 0.557400

c_group:c_age 20.07 20.07 1 166.60 0.1307 0.718132

c_direction:c_age 217.62 217.62 1 168.22 1.4178 0.235446

c_direction:c_group:c_age 702.63 702.63 1 168.22 4.5775 0.033836 *

Primary pulse accuracy

Type III Analysis of Variance Table with Satterthwaite's method

Sum Sq Mean Sq NumDF DenDF F value Pr(>F)

c_handtested 0.04762 0.04762 1 319.53 0.2214 0.638315

c_group 0.04197 0.04197 1 96.49 0.1951 0.659666

c_age 0.80304 0.80304 1 84.88 3.7336 0.056665 .

c_sex 0.96074 0.96074 1 95.41 4.4667 0.037171 *

c_location 2.78410 2.78410 1 94.11 12.9440 0.000514 ***

c_group:c_sex 0.36091 0.36091 1 96.73 1.6780 0.198278

c_handtested:c_group 0.00065 0.00065 1 319.92 0.0030 0.956159

c_handtested:c_sex 0.02098 0.02098 1 319.31 0.0975 0.754999

c_handtested:c_group:c_sex 0.25542 0.25542 1 319.95 1.1875 0.276652

Saccade gain

Type III Analysis of Variance Table with Satterthwaite's method

Sum Sq Mean Sq NumDF DenDF F value Pr(>F)

c_group 0.015609 0.015609 1 163.15 0.9980 0.31926

c_age 0.000546 0.000546 1 166.14 0.0349 0.85197

c_group:c_age 0.091803 0.091803 1 166.14 5.8697 0.01648 *

Force test: Coefficient of variation

Type III Analysis of Variance Table with Satterthwaite's method

Sum Sq Mean Sq NumDF DenDF F value Pr(>F)

c_MVC.pct 5.1374 2.56869 2 1963.75 227.6514 < 2.2e-16 ***

c_group 0.3349 0.33494 1 139.39 29.6842 2.238e-07 ***

c_age 0.4733 0.47334 1 140.15 41.9501 1.473e-09 ***

c_location 0.1336 0.13355 1 138.47 11.8361 0.0007682 ***

c_MVC.pct:c_group 0.3833 0.19166 2 1965.39 16.9858 4.855e-08 ***

c_MVC.pct:c_age 0.0243 0.01213 2 1964.55 1.0753 0.3413811

c_group:c_age 0.0220 0.02196 1 140.27 1.9462 0.1652009

c_MVC.pct:c_group:c_age 0.1447 0.07236 2 1964.39 6.4129 0.0016747 **

Gain test: Coefficient of variation

Type III Analysis of Variance Table with Satterthwaite's method

Sum Sq Mean Sq NumDF DenDF F value Pr(>F)

c_visual.angle 1.38469 0.69235 2 1450.56 59.9363 < 2.2e-16 ***

c_group 0.41654 0.41654 1 88.63 36.0600 4.126e-08 ***

c_age 0.36206 0.36206 1 89.73 31.3432 2.332e-07 ***

c_age_sq 0.17821 0.17821 1 88.48 15.4276 0.0001695 ***

c_visual.angle:c_group 0.25118 0.12559 2 1451.71 10.8724 2.057e-05 ***

c_visual.angle:c_age 0.12759 0.06380 2 1448.67 5.5227 0.0040795 **

c_group:c_age 0.08990 0.08990 1 88.15 7.7826 0.0064641 **

c_visual.angle:c_group:c_age 0.13566 0.06783 2 1450.21 5.8722 0.0028842 **

Saccade gain variability

Type III Analysis of Variance Table with Satterthwaite's method

Sum Sq Mean Sq NumDF DenDF F value Pr(>F)

c_direction 0.000658 0.000658 1 166.08 0.7037 0.4027497

c_age 0.014210 0.014210 1 167.13 15.1881 0.0001406 ***

c_location 0.049234 0.049234 1 167.34 52.6216 1.435e-11 ***

c_direction:c_age 0.002295 0.002295 1 166.18 2.4525 0.1192402

Force test: Approximate entropy

Type III Analysis of Variance Table with Satterthwaite's method

Sum Sq Mean Sq NumDF DenDF F value Pr(>F)

c_MVC.pct 4.0121 2.00605 2 1993.01 223.5369 < 2.2e-16 ***

c_handtested 0.0026 0.00260 1 1981.42 0.2896 0.5905190

c_group 0.1234 0.12342 1 131.61 13.7533 0.0003062 ***

c_age 0.2046 0.20464 1 129.53 22.8033 4.777e-06 ***

c_location 1.2143 1.21433 1 130.07 135.3141 < 2.2e-16 ***

c_sex 0.0074 0.00743 1 131.48 0.8278 0.3645722

c_age_sq 0.0655 0.06554 1 129.49 7.3037 0.0078039 **

c_MVC.pct:c_group 0.0256 0.01280 2 1993.64 1.4268 0.2403094

c_MVC.pct:c_age 0.1391 0.06956 2 1989.68 7.7509 0.0004435 ***

c_group:c_age 0.0016 0.00160 1 132.10 0.1788 0.6730838

c_MVC.pct:c_sex 0.0827 0.04134 2 1993.56 4.6069 0.0100889 *

c_group:c_sex 0.0001 0.00009 1 131.69 0.0098 0.9212607

c_MVC.pct:c_group:c_age 0.1128 0.05638 2 1989.86 6.2828 0.0019054 **

c_MVC.pct:c_group:c_sex 0.0653 0.03263 2 1993.40 3.6363 0.0265233 *

Gain test: Approximate entropy

Type III Analysis of Variance Table with Satterthwaite's method

Sum Sq Mean Sq NumDF DenDF F value Pr(>F)

c_visual.angle 0.78802 0.39401 2 1399.31 52.2662 < 2.2e-16 ***

c_handtested 0.00352 0.00352 1 1408.10 0.4673 0.4943670

c_group 0.16351 0.16351 1 97.74 21.6905 1.011e-05 ***

c_age 0.18730 0.18730 1 97.71 24.8463 2.689e-06 ***

c_sex 0.00046 0.00046 1 98.55 0.0611 0.8052104

c_visual.angle:c_sex 0.04923 0.02461 2 1397.93 3.2650 0.0384900 *

c_visual.angle:c_age 0.11711 0.05856 2 1401.09 7.7675 0.0004418 ***

c_group:c_age 0.00147 0.00147 1 97.61 0.1946 0.6600816

c_handtested:c_age 0.00071 0.00071 1 1405.45 0.0940 0.7592098

c_handtested:c_group 0.00000 0.00000 1 1408.78 0.0006 0.9800245

c_handtested:c_group:c_age 0.04520 0.04520 1 1405.80 5.9957 0.0144620 *
