# Supplementary material for: Initial action output and feedback-guided motor behaviors in autism spectrum disorder
Source: Mol Autism. 2021 Jul 10;12:52. doi: 10.1186/s13229-021-00452-8 (PMC8272343; doi:10.1186/s13229-021-00452-8)

**Force task**

Missing trials by age (< 16 and > 15), group, and condition:


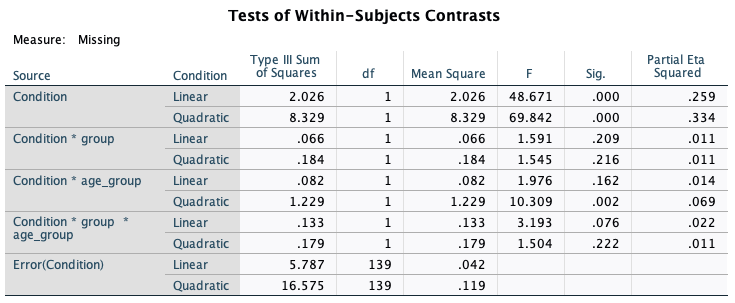


Simple effects for age group 1 (< 16 years):


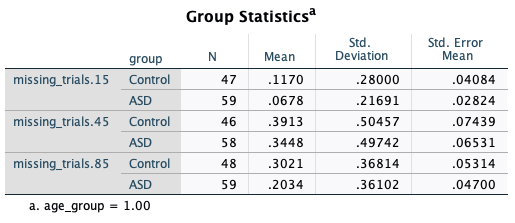

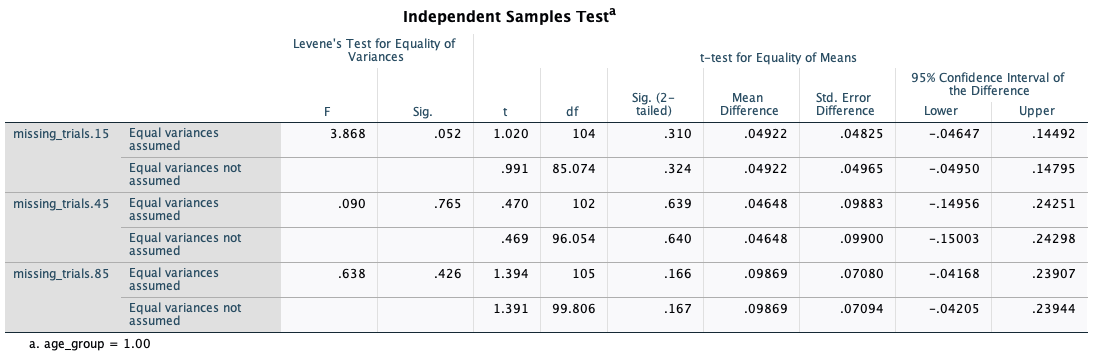


Simple effects for age group 2 (> 15 years):


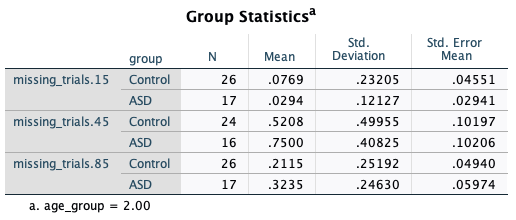


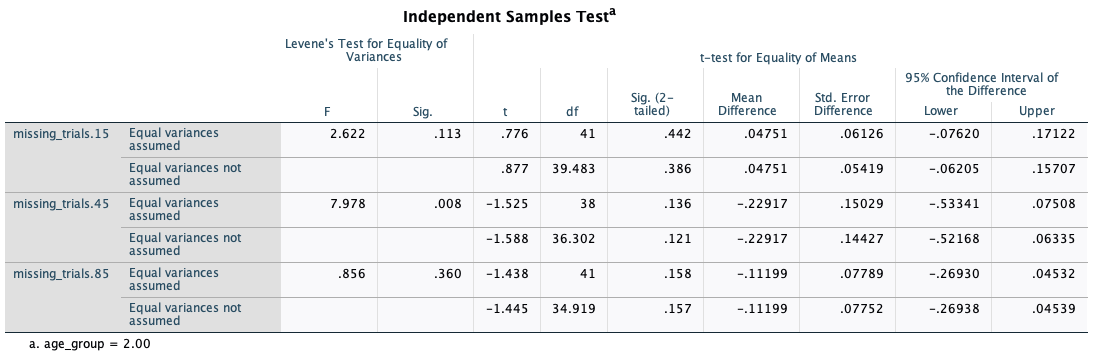


Missing trials by condition and location:


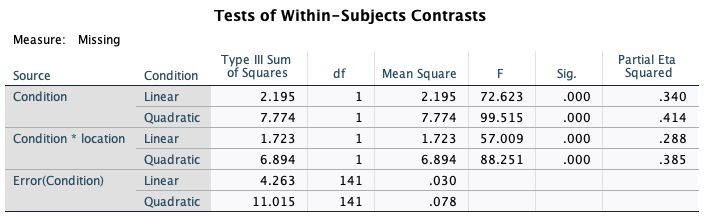


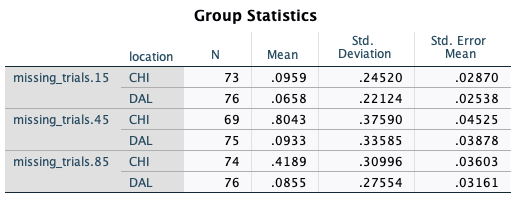


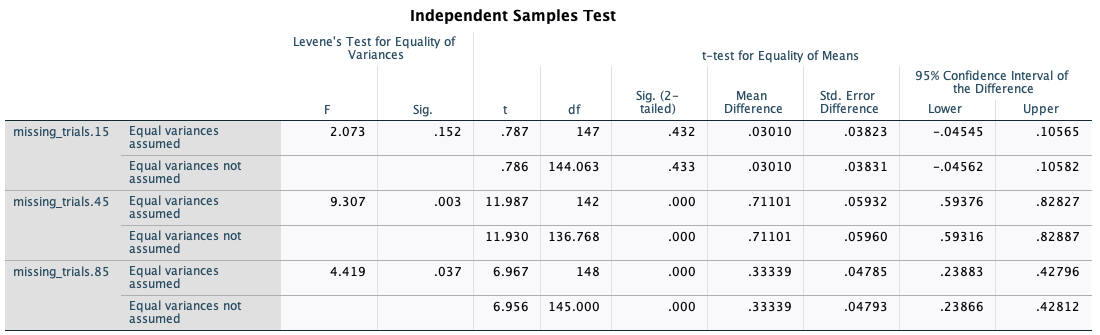


Average trial exclusion was greater for data collected at UIC (CHI) compared to UTSW (DAL) for 45% and 85% trials.

**Gain task**

Missing trials by age (< 16 and > 15), group, and condition:


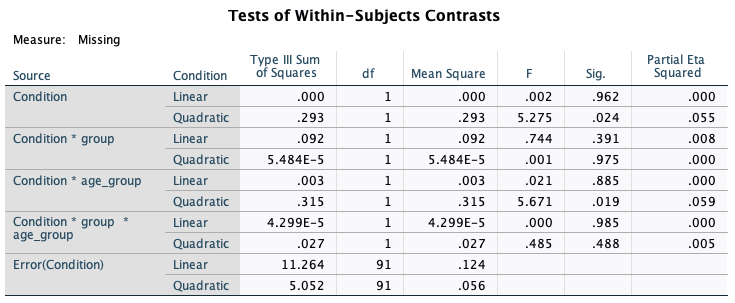


Simple effects for age group 1 (< 16 years):


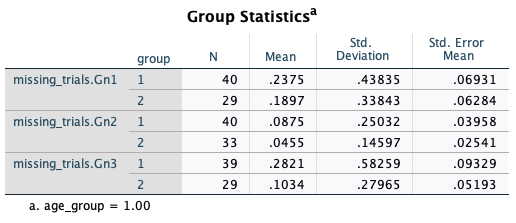


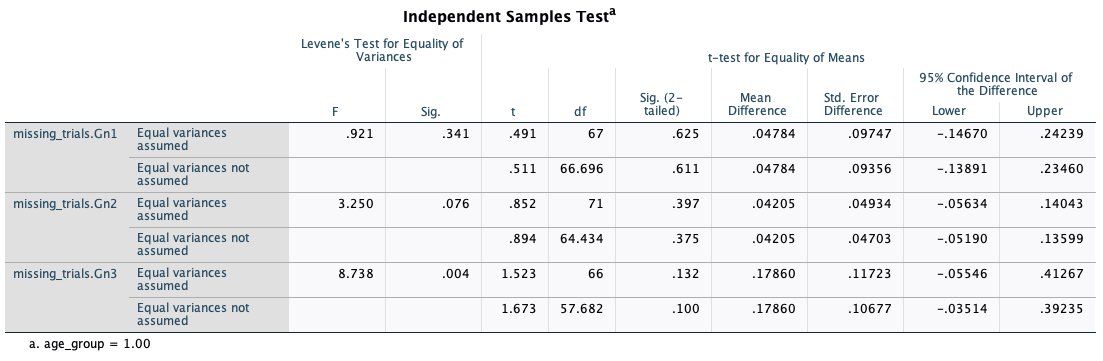


Simple effects for age group 2 (> 15 years):


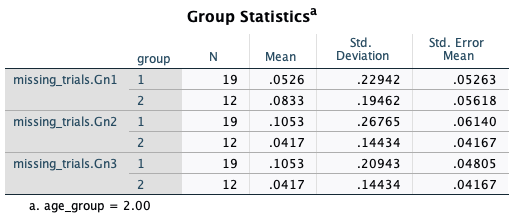


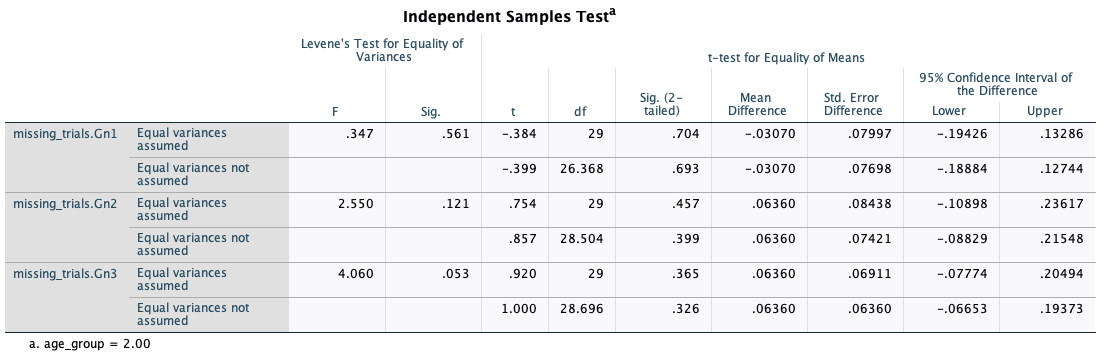


Missing trials by condition and location:


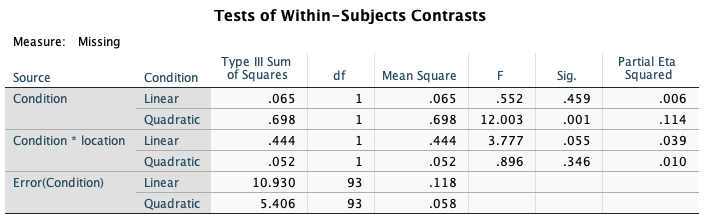


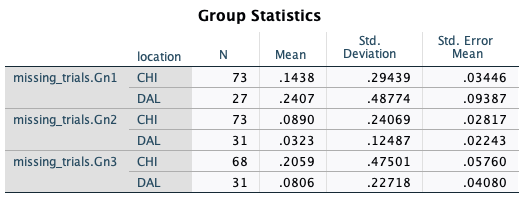


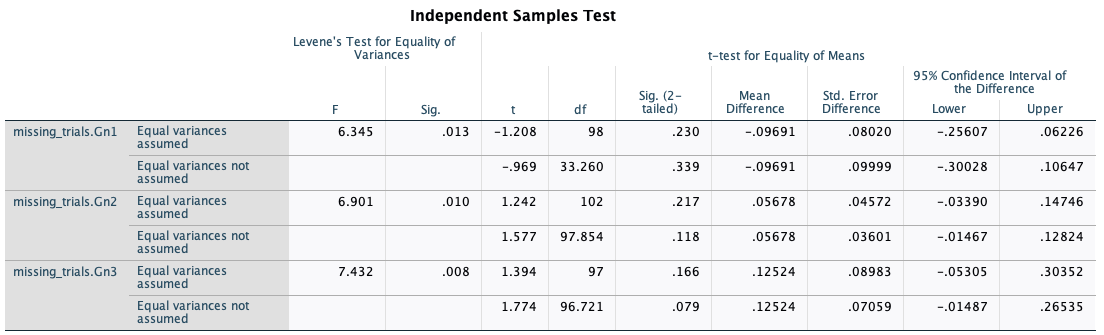


**VGS task**

Missing trials by age (< 16 and > 15) and group:


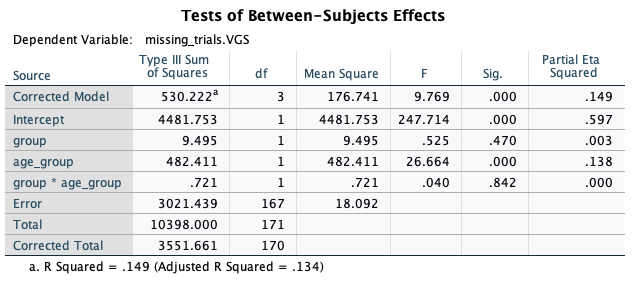


Simple effects for age group 1 (< 16):


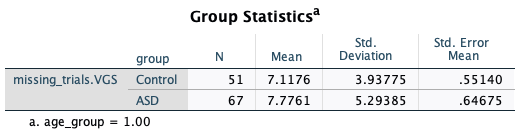


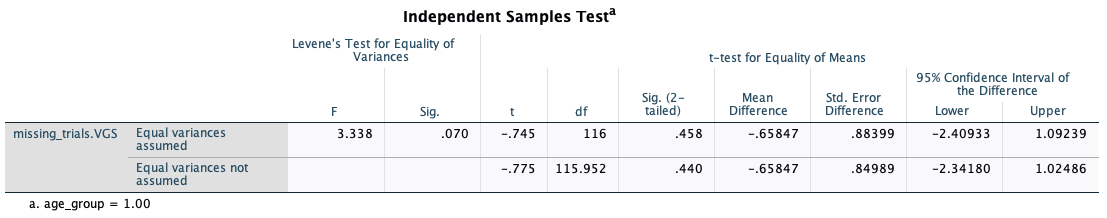


Simple effects for age group 2 (> 15):


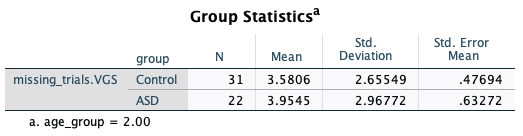


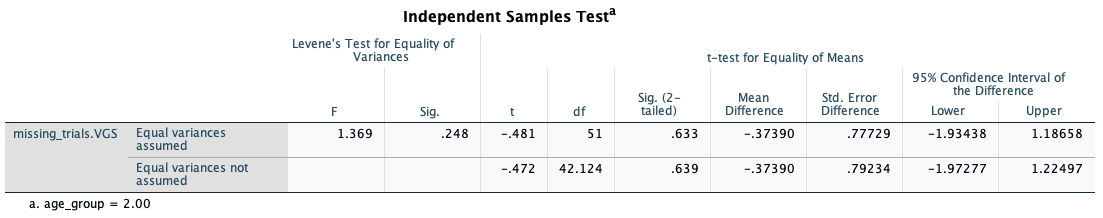


Missing trials by location:


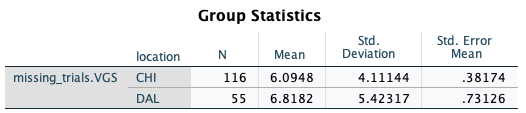


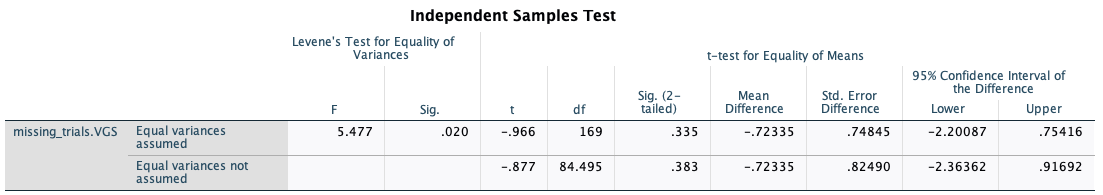

Supplement: Supplementary file 3 — Additional file 3. Linear mixed effects model results. [file 13229_2021_452_MOESM3_ESM.docx]
