# Supplementary material for: Initial action output and feedback-guided motor behaviors in autism spectrum disorder
Source: Mol Autism. 2021 Jul 10;12:52. doi: 10.1186/s13229-021-00452-8 (PMC8272343; doi:10.1186/s13229-021-00452-8)
Supplement: Supplementary file 4 — Additional file 4. Additional figures 1–4. [file 13229_2021_452_MOESM4_ESM.pdf]

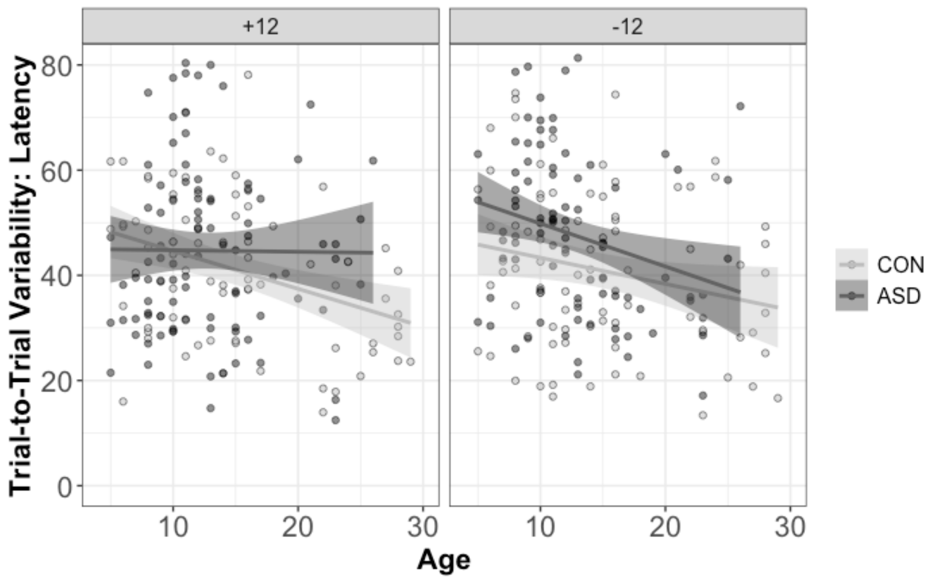

Supplementary Figure 1. Latency variability decreased with age in both groups for leftward saccades (-12 degree trials, group x age: $\beta = -0.32$, $t = -0.80$, $p = .42$). For rightward saccades, control participants again showed age-related reductions in latency variability ($\beta = -0.72$, $t = -3.16$, $p = .002$), while individuals with ASD did not ($\beta = -0.03$, $t = -0.09$, $p = .93$).

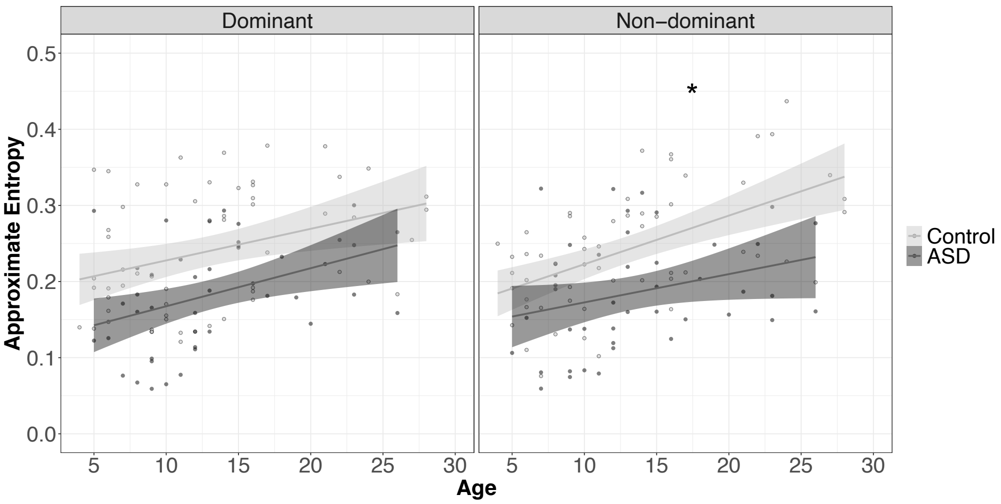

Supplementary Figure 2. Control participants show increased age-associated improvements in ApEn relative to ASD in the non-dominant hand only. Error bars reflect standard error of the mean. **slopes differ between groups*

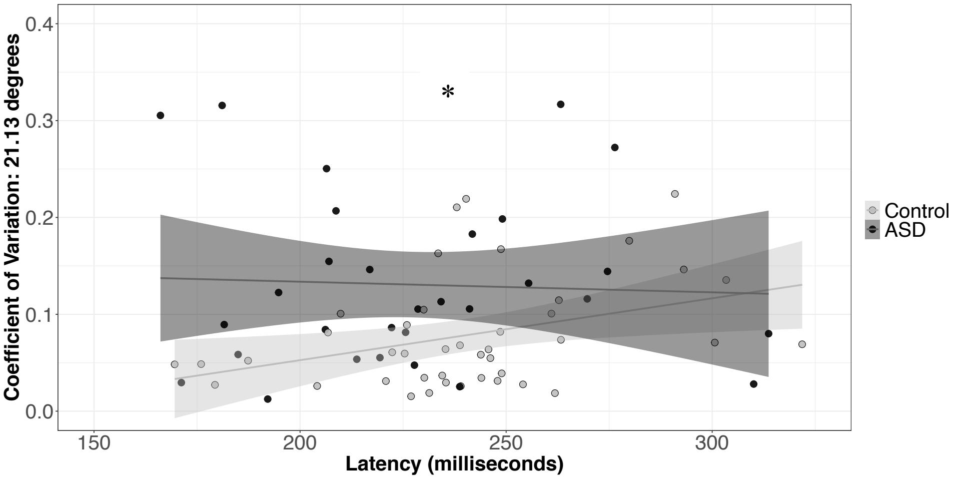

Supplementary Figure 3. Saccade latency was associated with increased CoV (21.13 degrees) in control participants, and the strength of this relationship was stronger in controls than in ASD (z = 2.00, p = .045). **slopes differ between groups*

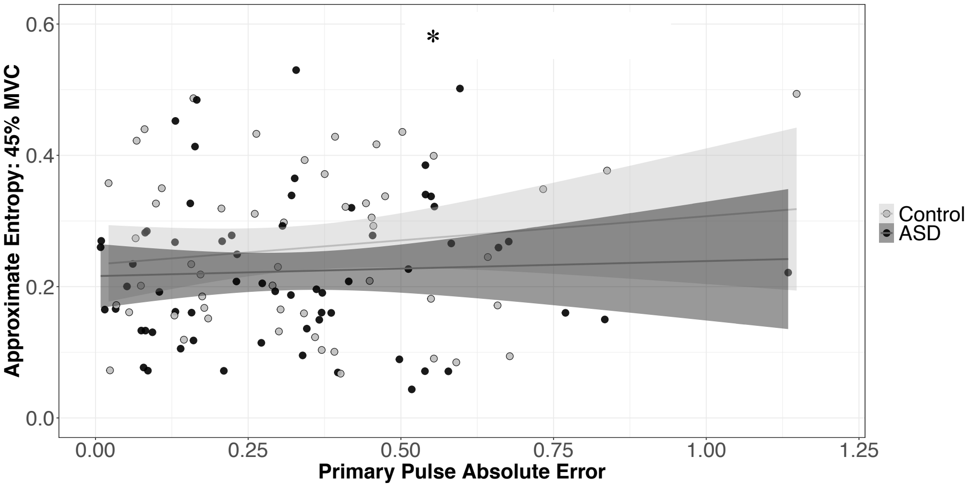

Supplementary Figure 4. In control participants, increased saccade error was associated with reduced ApEn (15, 45, 85% MVC) and the strength of this association was stronger in controls than in ASD (45%; z = -2.43, p = .015). **slopes differ between groups*
